# Biosynthesis of zinc oxide nanoparticles *via* neem extract and their anticancer and antibacterial activities

**DOI:** 10.7717/peerj.17588

**Published:** 2024-06-25

**Authors:** Hossam S. El-Beltagi, Marwa Ragab, Ali Osman, Ragab A. El-Masry, Khairiah Mubarak Alwutayd, Hind Althagafi, Leena S. Alqahtani, Reem S. Alazragi, Ahlam Saleh Alhajri, Mahmoud M. El-Saber

**Affiliations:** 1 Agricultural Biotechnology Department, College of Agriculture and Food Sciences, King Faisal University, Al-Ahsa, Saudi Arabia; 2 Biochemistry Department, Faculty of Agriculture, Cairo University, Giza, Egypt; 3 Biochemistry Department, Faculty of Agriculture, Zagazig University, Zagazig, Egypt; 4 Department of Biology, College of Science, Princess Nourah bint Abdulrahman University, Riyadh, Saudi Arabia; 5 Department of Biochemistry, College of Science, University of Jeddah, Jeddah, Saudi Arabia; 6 Food Science and Nutrition Department, College of Agricultural and Food Science, King Faisal University, Al-Ahsa, Saudi Arabia; 7 Biochemistry Unit, Genetic Resources Department, Desert Research Center, Cairo, Egypt

**Keywords:** Neem, ZnO-NPs, Green nanoparticles, Anticancer, Antibacterial, Phenolic compounds

## Abstract

In the present study, zinc oxide nanoparticles (ZnO-NPs) were synthesized using neem leaf aqueous extracts and characterized using transmission electron microscopy (TEM), ultraviolet visible spectroscopy (UV-Vis), and dynamic light scattering (DLS). Then compare its efficacy as anticancer and antibacterial agents with chemically synthesized ZnO-NPs and the neem leaf extract used for the green synthesis of ZnO-NPs. The TEM, UV-vis, and particle size confirmed that the developed ZnO-NPs are nanoscale. The chemically and greenly synthesized ZnO-NPs showed their optical absorbance at 328 nm and 380 nm, respectively, and were observed as spherical particles with a size of about 85 nm and 62.5 nm, respectively. HPLC and GC-MS were utilized to identify the bioactive components in the neem leaf aqueous extract employed for the eco-friendly production of ZnO-NPs. The HPLC analysis revealed that the aqueous extract of neem leaf contains 19 phenolic component fractions. The GC-MS analysis revealed the existence of 21 bioactive compounds. The antiproliferative effect of green ZnO-NPs was observed at different concentrations (31.25 µg/mL–1000 µg/mL) on Hct 116 and A 549 cancer cells, with an IC50 value of 111 µg/mL for A 549 and 118 µg/mL for Hct 116. On the other hand, the antibacterial activity against gram-positive and gram-negative bacteria was estimated. The antibacterial result showed that the MIC of green synthesized ZnO-NPs against gram-positive and gram-negative bacteria were 5, and 1 µg/mL. Hence, they could be utilized as effective antibacterial and antiproliferative agents.

## Introduction

Nanotechnology has become a promising technique in medicinal sciences, energy generation, nanoelectronics, and consumer products (Rajendran & Mani, 2020). Nanoparticle-based therapy is proposed as an alternate solution to address global challenges like antimicrobial resistance (AMR) and cancer (Mariappan et al., 2021). AMR is a significant worldwide public health issue, jeopardizing the efficacy of antibiotic treatment and creating obstacles to the development of new antibiotics. The incidence of morbidity and mortality resulting from drug-resistant diseases is increasing globally (Serwecińska, 2020). For this reason, the search for new antimicrobials from natural sources is crucial in modern medicine to combat the socioeconomic impact and health consequences of multidrug-resistant bacteria (Mahgoub, Sitohy & Osman, 2013; El-Beltagi et al., 2019; Ebrahim et al., 2022; Fekry et al., 2022). Consequently, the advancement of newer antimicrobial medications necessitates the adoption of a creative approach (Abdel-Shafi et al., 2016; Abdel-Shafi et al., 2019a; Abdel-Shafi et al., 2019b; Abdel-Hamid et al., 2020b; Al-Mohammadi et al., 2020; Osman et al., 2021a; Abdel-Shafi et al., 2022; Enan et al., 2023).

Another major disease and leading cause of death that has long been burdening the human population is cancer. Cancer is a prevalent disease caused by genetic abnormalities in cellular DNA, disrupting the process that controls cell death and division, resulting in uncontrollable cell growth in the human body (Yadav & Mohite, 2020). Traditional cancer therapies, including surgery, radiation, and chemotherapy, are successful but have significant side effects that diminish patients’ quality of life (Schirrmacher, 2019; Chandrasekaran, Anusuya & Anbazhagan, 2022). Plant-based medicines can mitigate the harmful side effects of cancer treatment (Abd Elhamid et al., 2022).

Free radicals such as reactive oxygen species and/or reactive nitrogen species (ROS/RSN) generated during metabolic pathways cause oxidative stress (Afify & El-Beltagi, 2011; Amer et al., 2022a; Amer et al., 2022b). Oxidative stress is linked to chronic and degenerative diseases such as cancer, autoimmune and age-related disorders, cataracts, rheumatoid arthritis, cardiovascular and neurodegenerative diseases (Abdel-Hamid et al., 2020a; Imbabi et al., 2021; Osman et al., 2021b; Reda et al., 2023).

However, this process can be regulated by antioxidants produced naturally or externally supplied through foods and herbal supplements such as bioactive peptides (Abdel-Rahim & El-Beltagi, 2010; Abdel-Hamid et al., 2017; Osman et al., 2019; El-Beltagi et al., 2020).

Metal nanoparticles, including zinc oxide nanoparticles, have been effectively produced using environmentally friendly methods for a variety of uses, frequently demonstrating superior characteristics compared to those produced using traditional synthetic methods (Schwartz, Marsh & Draelos, 2005; Iravani, 2011). Zinc oxide nanoparticles (ZnO-NPs) are gaining significance in the healthcare sector due to their various benefits. Distinct antibacterial and wound healing properties, UV filtering capability, and strong catalytic and photochemical effects (Nadhman et al., 2014). The biosynthesis of ZnO-NPs by plants and fungi has been reported with antibacterial and antifungal properties (Gunalan, Sivaraj & Rajendran, 2012; Najafi-Taher et al., 2018). Several chemical and biological techniques have been published for the synthesis of ZnO nanoparticles (NPs). In general, the most critical problem concerning the use of NPs in biomedical and biotechnological applications is the toxicity derived from the reducing agents and stabilizers used for the NP synthesis (*e.g.*, sodium borohydride, hydrazine, cetyltrimethylammonium bromide, various polymers Hone et al., 2002). To solve this problem, numerous researchers have concentrated on developing bio-friendly reagents for nanomaterial fabrication. Various natural compounds are being evaluated as nontoxic reducing agents or particle-surface stabilizers. Several natural compounds of plant origin (phytochemistry) are known to have anti-oxidant, anti-bacterial, and anti-inflammatory properties, as well as the ability to induce cell death in malignant cells (Bellik et al., 2012). However, green synthesis is one of the most environmentally friendly methods available (Schwartz, Marsh & Draelos, 2005; Iravani, 2011). Green synthesis of nanoparticles provides an advantage over other approaches since it is straightforward, one-step, cost-effective, environmentally friendly, and frequently results in more stable compounds (Ahmed et al., 2016).

*Azadirachta indica*, also known as neem, is highly valued in traditional Indian medicine due to its therapeutic benefits and rich phenolic content (Kumar & Navaratnam, 2013). *A. indica* has been used for centuries to treat a variety of ailments including inflammation, diarrhea, bacterial infections, and constipation. Its different parts, such as leaves, flowers, seeds, fruits, roots, and bark, have all been used for their therapeutic properties (Duangjai et al., 2019; Ramadan et al., 2022a; Ramadan et al., 2022b), cancer (Paul, Prasad & Sah, 2011), fever, and skin diseases (Al Saiqali et al., 2018). Additionally, *A. indica* has a wide range of pharmacological properties owing to its complex composition, which includes over 300 distinct bioactive chemicals with diverse activity (Gupta et al., 2017; Abdel Rahman et al., 2023; Rahman et al., 2023). The neem leaf extract, which contain functional substances such as cyclic peptides, sorbic acid, citric acid, phenolic compounds, polyhydroxy limonoids, ascorbic acid, retinoic acid, tannins, ellagic acid, and gallic acid, are thought to play an important role in the bioreduction and stabilisation of nanoparticles (Narde et al., 2023). Neem leaf extract contains phytochemicals such as flavones, organic acids, ketones, amides, and aldehydes. Flavones and organic acids, which are water-soluble, function as bio reductants and reduce zinc ions to form zinc nanoparticles (Sangeetha, Rajeshwari & Venckatesh, 2011).

Neem leaf extract contains phytochemicals such as flavones, organic acids, ketones, amides, and aldehydes. Flavones and organic acids, which are water-soluble, function as bio reductants and reduce zinc ions to form zinc nanoparticles (Sangeetha, Rajeshwari & Venckatesh, 2011). The main target of the present study was the green synthesis of ZnONPs *via* neem leaf aqueous extract and then comparing its efficacy as anticancer and antibacterial agents with chemically synthesized ZnONPs and the neem leaf extract used for the green synthesis of ZnONPs.

## Materials & Methods

### Plant materials

The leaves of *Azadirachta indica* A. Juss used in this study were provided by the Desert Research Centre (DRC) in Cairo, Egypt.

### Chemicals

DPPH (2,2-diphenyl-1-picrylhydrazyl; CAS Number: 84077-81-6), methanol; CAS Number: 67-56-1, zinc nitrate hexahydrate; CAS Number: 10196-18-6, sodium hydroxide; CAS Number: 1310-73-2, ethanol; CAS Number: 64-17-5, ethyl acetate; CAS Number: 141-78-6, gallic acid; CAS Number: 149-91-7, quercetin; CAS Number: 117-39-5, AlCl_3_; CAS Number: 7446-70-0, Folin-Ciocâlteu reagent; Mfcd00132625, sodium carbonate; CAS Number: 497-19-8, and potassium acetate; CAS Number: 127-08-2 were acquired from Merck (KGaA, Darmstadt, Germany).

### Extract preparation

The neem leaves were gathered and washed thoroughly with distilled water and both surfaces of the leaves were sterilized using alcohol by gentle rubbing to avoid any contamination. The leaves were subsequently dehydrated for a period of three days under direct exposure to sunshine. The process involves pulverizing and filtering dried leaves using a mechanical blender (High-Capacity Upgraded Version ABS Mechanical Blender, 30,000 piece/pieces per month; Ningbo, China). The water extract of neem leaves is prepared by dissolving 10 gm of leaves in 100 mL of distilled water. The mixture is then heated on a hot plate magnetic stirrer (Benchmark H3770-HS Digital Hotplate Stirrer, temps up to 380 °C and speeds 150 to 1,500 rpm. Probe with direct feedback to microprocessor) to a temperature of 50 °C for 30 min. Afterward, the solution is filtered using filter paper (Whatman No. 1, pore size of 11 µm) and subjected to centrifugation (Sigma 2-6 Benxhtop Centrifuge) at a force of 5,000× g for 15 min. Immediately after that, zinc oxide NPs was prepared using aqueous extract of neem. The stock solution was stored in the refrigerator at 4 °C. This solution was also used for the investigation of neem phytochemistry (Elumalai et al., 2012; El-Saber, 2021; Elshafie et al., 2023).

### Chemically synthesis of ZnO NPs

Zinc oxide nanoparticles were synthesized using a chemical process using zinc nitrate and sodium hydroxide as starting materials, whereas sodium borohydride has been used as a stabilizing agent, as described by Ahamed & Kumar (2016). In this experiment, a 0.1M solution of zinc nitrate (Zn (NO_3_)_2_.6H_2_O) in water was stirred well continuously for one hour using a magnetic stirrer (Hotplate Magnetic Stirrer LHST-A11) to ensure complete dissolution of the zinc nitrate. Similarly, a 0.8M solution of NaOH in water was also stirred consistently for one hour. Once the zinc nitrate had fully dissolved, a 0.8M aqueous solution of NaOH was added gradually, drop by drop, over a period of 45 min manually, and then 1 ml NaBH4 (1%) was added dropwise after zinc nitrate dissolution. The solution was continuously stirred at a high speed during this process. The NaOH was completely added to the reaction, and then the reaction was allowed to proceed for aduration of 2 h. The beaker remained sealed in this condition for 4 h by aluminum foil at room temperature. Once the reaction was finished, the solution was left undisturbed overnight before the supernatant solution was meticulously separated. The remaining solution was subjected to centrifugation at 5,000× g for 10 min to eliminate the precipitate. The precipitate was then dried in an oven at 80 °C. Zn(OH)_2_ was formed and then converted to ZnO. The calcination had been performed at 500 °C for 1 h. The optical and nanostructured characteristics of the produced ZnO NPs nanoparticles were investigated.

### Green synthesis of ZnO NPs by neem leaves aqueous extract

The synthesis of ZnO-NPs was accomplished through the combination of 10 ml of neem leaf extract (as the reducing and capping agents) in water with 90 ml of a solution containing 1 mM zinc nitrate hexahydrate [Zn (NO_3_)_2_.6H_2_O]. The mixture was heated on a hotplate magnetic stirrer at a temperature of 80 °C for 30 min, with continuous stirring. The existence of white-colored particles indicated the formation of nanoparticles. The particles obtained were subjected to centrifugation, and the resulting solid masses were gathered. The pellet was dried and calcined at 300 °C for 2 h. The process was followed to obtain white colored nanoparticles (Pal et al., 2018; Zambri et al., 2019; El-Saber, 2021; Elshafie et al., 2023).

### Green and chemically synthesized ZnO-NPs characterization

### Transmission electron microscopy

Actual morphology of the as-prepared ZnO-NPs was imaged by High-resolution transmission electron microscopy (HR-TEM) operating at an accelerating voltage of 200 kV (Tecnai G2; FEI). Diluted ZnO NPs suspension was ultra-sonicated for 5 min to reduce the particles aggregation. Using micropipette, about three drops from the ultra-sonicated solution were deposited on carbon coated-copper grid (200 mesh) and left to dry at room temperature. HR-TEM images of the ZnO NPs that were deposited on the grid were captured for morphological evaluation. All the preparation and characterization processes were conducted at the Nanotechnology and Advanced Materials Central Lab (NAMCL), Agricultural Research Center, Egypt.

### Ultraviolet-Visible (UV-VIS) spectra

The Shimadzu spectrophotometer, namely the UV-VIS module (UV-2450; Shimadzu), was employed to track the progression of ZnO-NPs formation inside the aqueous solution containing neem extract. The UV-Vis spectra were measured within the wavelength range of 300 to 700 nm.

### Dynamic Light Scattering (DLS), Zeta potential and particle size analysis

The average particle size distribution was determined using the DLS method, employing the zeta sizer (Malvern, ZS Nano, UK). The solution sample was placed in a cuvette (ZEN2112 Low-volume Quartz cuvette 20 µL) and treated until it became transparent, reducing the error in the reading. The device then measured the diameter of particles and determined the level of homogeneity using the PDI (dispersed index), which ranges from 0 to 1. A PDI approaching zero indicates increased homogeneity.

### Qualitative phytochemicals analysis of neem leaves aqueous extract

Following standard methods, preliminary qualitative phytochemical screening was performed (Shrestha et al., 2015).

### Alkaloids detection

The alkaloids were tested using Mayer’s reagent (freshly prepared by dissolving a mixture of mercuric chloride (1.36 g) and potassium iodide (5.00 g) in 100 ml distilled water). A volume of two mL of botanical extract was introduced into a test tube, followed by the addition of 2–3 drops of Mayer’s reagent. The detection of an alkaloid was indicated by the production of a green precipitate in the solution. Wagner’s test was conducted utilizing Wagner’s reagent (potassium iodide (2 g) and iodine (1.27 g) were dissolved in distilled water (5 mL) and the solution was diluted to 100 mL with distilled water). The presence of alkaloids was shown by the formation of a reddish-brown precipitate in a test tube holding 2 ml of extract upon addition of a few drops of Wagner’s reagent.

### Flavonoids detection

A total of two mL of plant material was added to a test tube, along with an equal volume of a NaOH solution containing 2% w/v. A vivid yellow hue manifested within the test tube. The addition of a small amount of dilute hydrochloric acid resulted in the loss of colour, showing the existence of flavonoids. A volume of two mL of botanical extract was introduced into a test tube in preparation for the Shinoda Test. The substance underwent treatment with 5 drops of hydrochloric acid and 0.5 grammes of magnesium bits. The solution, which contained flavonoids, had a pink hue.

### Terpenoids detection

The obtained extract was dissolved in two mL of chloroform and subjected to evaporation until complete dryness. A volume of two mL of concentrated H_2_SO_4_ was introduced into the mixture. The presence of terpenoids is evidenced by the appearance of a reddish-brown colour at the boundary between the two substances.

### Saponins detection

Foam test was used to detect saponin. The concentrated solution was diluted with distilled water and transferred into a test tube. There was a temporary cessation for a few minutes. Saponins were detected within a foam layer of two cm in thickness.

### Steroids detection

The obtained extract was combined with chloroform (two mL), and then concentrated H_2_SO_4_ was added on the side. The manifestation of a crimson hue in the lower chloroform stratum signals the detection of steroids. Another experiment was carried out by combining the crude extract with two mL of chloroform. The mixture was then treated with 2 ml of concentrated H_2_SO_4_ andacetic acid. Steroid presence is signaled by the emergence of a green hue.

### Cardiac glycosides

Salkowski’s test involved the combination of 2ml of crude extract with two mL of chloroform. The next step was a cautious addition of two mL of concentrated H_2_SO_4_, then gentle mixing. The presence of a reddish-brown color indicates the presence of a steroidal ring, specifically the aglycone portion of the glycoside.

The Keller-Kilani test involved mixing a crude extract with two mL of glacial acetic acid that contained 1–2 drops of a 2% FeCl_3_ solution. The liquid was transferred into a separate test tube containing two mL of concentrated H_2_SO_4_. The presence of cardiac glycosides can be inferred from the existence of a brown ring at the contact.

### Polyphenols and tannins

The obtained extract was mixed with two mL of a 2% FeCl_3_ solution. The blue–green or blue-black color suggested the presence of polyphenols and tannins.

### Total phenolic compounds determination

Total phenolic compounds (TPCs) were determined using the Folin–Ciocalteau method, according to the method of Sánchez-Rangel et al. (2013) with some modifications. The 0.3 mL from neem aqueous extract (500 µg/mL) was added and mixed with 1.2 mL of Folin and Ciocalteu’s reagent (previously diluted 10-fold with distilled water). After 3 min, 1.5 mL of saturated Na_2_CO_3_ (75%) was subsequently added to the mixture. The samples were then incubated at 50 °C for 1 h, cooled and read at 765 nm. A calibration curve was created using a standard solution of gallic acid.

\begin{eqnarray*}y=0.001x+0.0563~{R}^{2}=0.9792 \end{eqnarray*}

where *y* represents absorbance and *x* represents gallic acid concentration in µg/mL.

All results were expressed as milligram of gallic acid per gram of extract (mg GAE/g extract).

### Total flavonoid content estimation

Total flavonoids content (TFC) in neem aqueous extract were analyzed with aluminum chloride method (Ordoñez et al., 2006) with a slight change. The assay mixture consisting of 0.5 mL of the neem aqueous extract (1,000 µg/mL), 0.5 mL distilled water, and 0.3 mL of 5% NaNO_2_ was incubated for 5 min at 25 °C. This was followed by the addition of 0.3 mL of 10% AlCl_3_ immediately. Two milliliters of 1 M NaOH was then added to the reaction mixture, and the absorbance was measured at 415 nm. The standard curve was created using quercetin. The total flavonoid levels were calculated using the calibration curve and the quercetin equivalent (QE).

\begin{eqnarray*}y=0.0012x+0.008~{R}^{2}=0.944 \end{eqnarray*}

where *x* is the quercetin concentration in µg/mL and *y* is the absorbance.

All results were expressed as milligram of quercetin per gram of extract (mg QE/g extract).

### Antioxidant activity estimation (DPPH-assay)

The antioxidant activity of the neem aqueous extract was assessed using the DPPH test method outlined by Ramadan, Osman & El-Akad (2008). Briefly, 2.9 mL of a 0.1 mM DPPH methanolic solution was combined with each extract concentration (500, 1,000, 1,500, or 2,000 µg/mL). The reaction proceeded in dark for 30 min at 25 °C. The absorbance of the combination was measured at 517 nm. The radical scavenging ability of DPPH was determined using the subsequent formula:

\begin{eqnarray*}\text{Inhibition}(\%)=[(\text{Control Abs}.-\text{Sample Abs}.)/\text{Control Abs}.]\times 100. \end{eqnarray*}

where Abs. control is the control absorbance and Abs. sample is the absorbance in the presence of extract.

### Phenolic compounds identification

High-performance liquid chromatography (HPLC) was used to identify polyphenolics. Compounds in the neem aqueous extract (Abd Elhamid et al., 2022). The phenolic compound detection was performed using an Agilent 1,260 Infinity HPLC system (Agilent, Santa Clara, CA, USA). The system was equipped with a quatpump (G 1311C), autosampler (G 1329B), column heater (G 1316A), variable wavelength detector (G 1314F), and an online degasser (G 1322A). The Agilent HPLC ChemStation 10.1 edition was utilized on a Windows 7 operating system to oversee instruments and conduct data analysis. The column used was an Agilent Zorbax C18 column with dimensions of 5 m in length and 4.6 mm in diameter, and a total length of 150 mm. The injection volume was 50 µL, and the Mobile Phase consisted of two components: A, which was a mixture of 70% methanol and 30% water, and B, which was 100% methanol. Samples were quantified by comparing the retention times with known authentic standards.

### Gas chromatography-mass spectrometry (GC-MS) analysis

Lyophilized neem crude extract (0.1 gm) was dissolved in 10 mL ethyl acetate. The neem ethyl acetate extract was examined *via* the GC-MS Agilent Technologies-7820A GC equipment. The capillary column used is the Agilent Technologies GC-MS HP-5MS, which has a length of 30 m, an inner diameter of 0.25 mL, and a film thickness of 0.25 m. The column is made of a 5% diphenyl and 95% dimethyl polysiloxane mixture. It is connected to the Agilent Technologies GCMS mass spectrometer model 5977MSD. A 70-eV electron ionization device was used. The carrier gas used was helium gas with a purity of 99.99%. The split ratio was 50:1, the injection volume was one mL, the injector temperature was set at 60 °C, and the ion source temperature was set at 250 °C. The temperature of the transfer line and ion source was set to 240 °C. The ionization mode used was electron impact at 70 eV. The scan time and scan interval were set to 0.2 s and 0.1 s, respectively.

The fragment size range is 40–600 Da. The components in the extracts were originally identified using peaks in the mass spectra utilizing Computer Wiley MS libraries, and these identifications were verified by comparing the two sets of data (Balamurugan, Balakrishnan & Sundaresan, 2015).

### Anticancer activity estimation

### Cell viability *in vitro* (MTT-assay)

The cell lines HCT116 (human colorectal cancer), and A549 (adenocarcinomic human alveolar basal epithelial cells), were obtained from Merck (KGaA, Darmstadt, Germany). The cells were sub-cultured in DMEM medium (Sigma-Aldrich) supplemented with 10% heat-inactivated foetal bovine serum (FBS), penicillin (10 U/mL), and streptomycin (10 µg/mL). The cultures were kept in an incubator at 37 °C, 5% CO_2_, and 100% humidity. 10 × 10^3^ cells were put into each well of a 96-well microplate, and the cells grew for 24 h at 37 °C and 5% CO_2_ before the samples were added. Different amounts (31.25–1000 µg/mL) of ZnO-NPs, neem extract, and green synthesised ZnO-NPs dissolved in distilled water were used to treat the cells. After 48 h of incubation, the absorbance at 550 nm was used to measure cell viability with the colorimetric MTT test (Promega, Madison, WI, USA) (Hansen, Nielsen & Berg, 1989). The positive control consisted of cells treated with a known volume of Triton X-100 (10 µL of a 10% solution), while the negative control consisted of cells left untreated. Percentages of cell viability and cytotoxicity were determined using the following equations:

\begin{eqnarray*}\text{Cell viability}(\%)=(\text{Ab sample}/\text{Ab control})\times 100 \end{eqnarray*}

\begin{eqnarray*}\text{Cytotoxic activity}(\%)=100\%-\text{cell viability}(\%). \end{eqnarray*}

The IC_50_ value describes the concentration of a sample that results in a 50% reduction in growth.

### The quantification of caspase-9 mRNA expression

The Step-One Plus Real-time PCR, manufactured by Applied Biosystems in Foster City, CA, USA, was utilized to quantitatively analyze caspase-9 in two human cancer cell lines—A549 and HCT116. This analysis was carried out before and after treatments with ZnO-NPs, neem aqueous extract, and green synthesized ZnO-NPs, using gene-specific primers and SYBR Green master mix. The primers were designed using Oligo 7 software and tested for accuracy and specificity on the NCBI website. Cells were treated with different samples’ IC_50_ for 24 h. Quantitative real-time PCR was employed to assess the relative expression level of caspase 9. The average score of duplicated Ct values was measured for each sample, and the relative expression levels of the target genes were determined using the comparative Ct method. The caspase 9-forward and caspase 9-reverse primer sequences were 5′-GCAGGCTCTGGATCTCGGC-3′ and 5′-GCTGCTTGCCTGTTAGTTCGC-3′, respectively. The annealing temperatures for these primers were 60.5 and 59.5 °C, respectively (Asadi et al., 2018).

### Antibacterial activity

### Minimum inhibitory concentration estimation

The minimum inhibitory concentration (MIC) of neem aqueous extract, ZnO-NPs, and green synthesized ZnO-NPs against gram-positive (*Listeria monocytogens* and *Staphylococcus aureus*) and gram-negative (*Salmonella Enteritidis* and *Escherichia coli*) bacteria was determined using the microdilution method. To experiment, 20 µL of 24-hour-old bacterial culture from well number 1 was added to each well of a 96-well plate. Following that, 100 µL of the tested samples were put into each well along with the same amount of Mueller-Hinton broth (MHB). The concentrations were 20, 10, 5, 2, 1, and 0.5 µg/mL. To serve as a control, a solution devoid of particles was utilized. The plates were left to incubate for the duration of the night. A 4% w/v p-iodonitro-tetrazolium violet solution (INT, Sigma-Aldrich) was added to 20 µL of each well to show that bacteria were present. The MIC was defined as the lowest concentration of the sample that effectively inhibited the development of microorganisms (Tayel et al., 2010).

### Transmission electron microscopy

*Staphylococcus aureus* and *Escherichia coli* were cultivated in Mueller Hinton Broth (MHB) and incubated at 37 °C until they reached a maximum concentration of 10^9^ CFU mL^−1^. The culture was then diluted to 10^8^ CFU mL^−1^ using a peptone solution (0.1% with 0.85% NaCl). The neem aqueous extract, ZnO-NPs, and green-synthesised ZnO-NPs were added to the cell suspensions, except for the control, and incubated at 37 °C for 4 h. Bacterial cells were collected, fixed, washed, and dehydrated before examination using a transmission electron microscope (JEOL-TME-2100F) as described by Sitohy et al. (2013).

### Statistical analysis

The recorded data was analyzed using version 21 of the Statistical Package for Social Sciences (SPSS), developed by SPSS Inc. (Chicago, IL, USA). The analysis was conducted following the guidelines provided (Dytham, 2011). The parameters were subjected to a one-way analysis of variance (ANOVA) test and the Duncan test to assess the statistical significance of the differences in means. Data were deemed statistically significant if the *P* ≤ 0.05.

## Results

### Characterization of ZnO-NPs prepared chemically and green synthesis

### Characterization of ZnO-NPs prepared chemically

**Figure 1:**
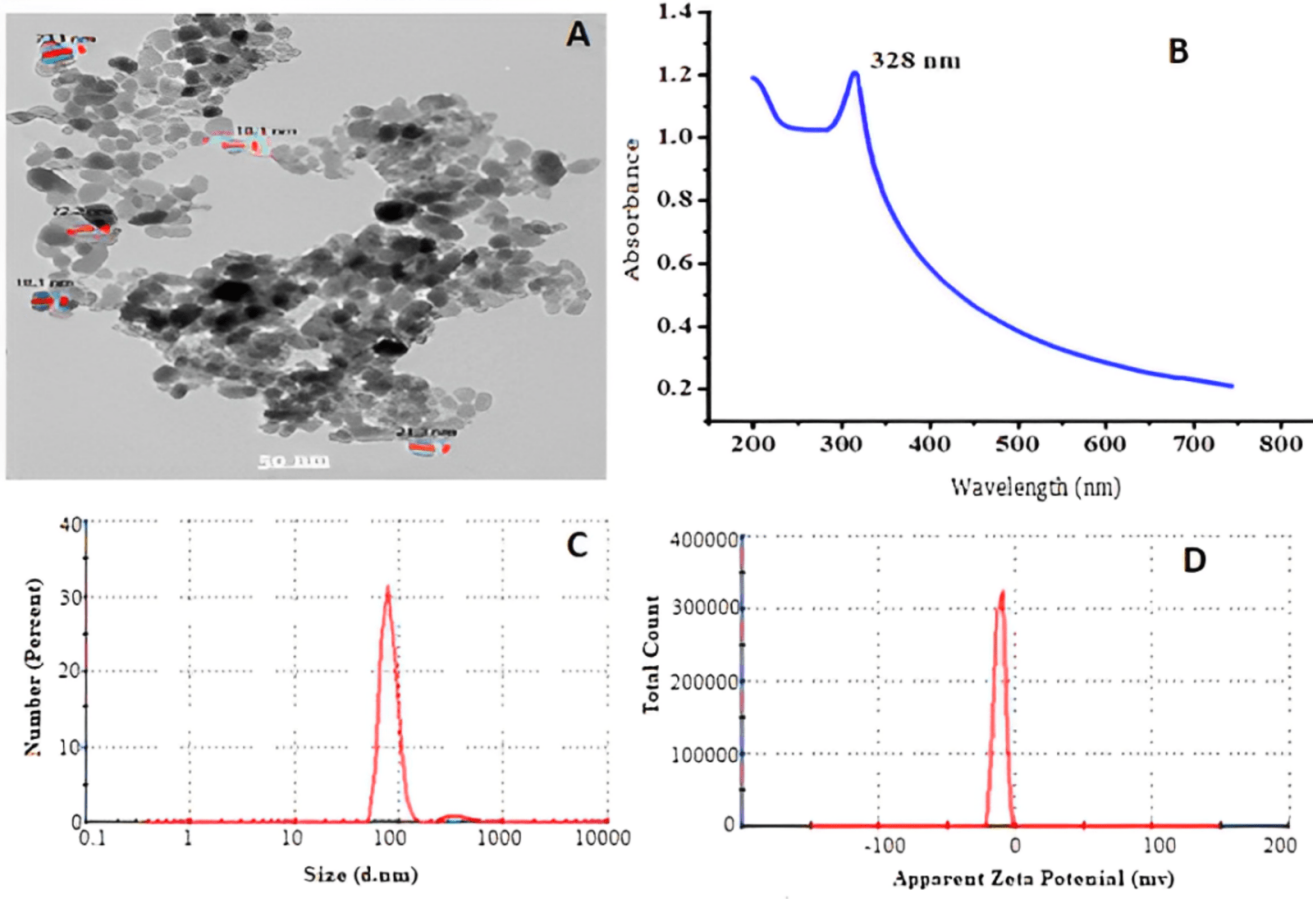
Transmission electron microscope (A), absorbance of ZnO-NPs UV-VS (B) particle size (C), and zeta potential (D) of ZnO NPs prepared using chemical method.

The analysis of the generated ZnO-NPs showed that they were disseminated in an essentially spherical shape with a particle size of approximately 85 ± 5 nm, as observed using transmission electron microscopy (TEM), as shown in Fig. 1A. UV-VIS optical absorption of ZnO-NPs. Figure 1B displays the UV-VIS absorption spectrum of ZnO-NPs. The absorbance spectra of the colloidal ZnO NPs solution displays a surface Plasmon resonance peak at 328 nm, indicating successful production and dispersion of the nanoparticles in the aqueous solution without any aggregation. In Fig. 1C, DLS was used for additional confirmation to assess the uniformity and estimate the average particle size, which was found to be 85 ± 5 nm. The polydisperse index (pdi) of 0.552 indicates a limited range of sizes. Based on Fig. 1D, the Zeta potential is measured to be −11.5 mV, indicating that the NPs are somewhat stable in terms of their stability and surface charge.

### Characterization of ZnO-NPs green synthesis by aqueous neem leaves extract

**Figure 2:**
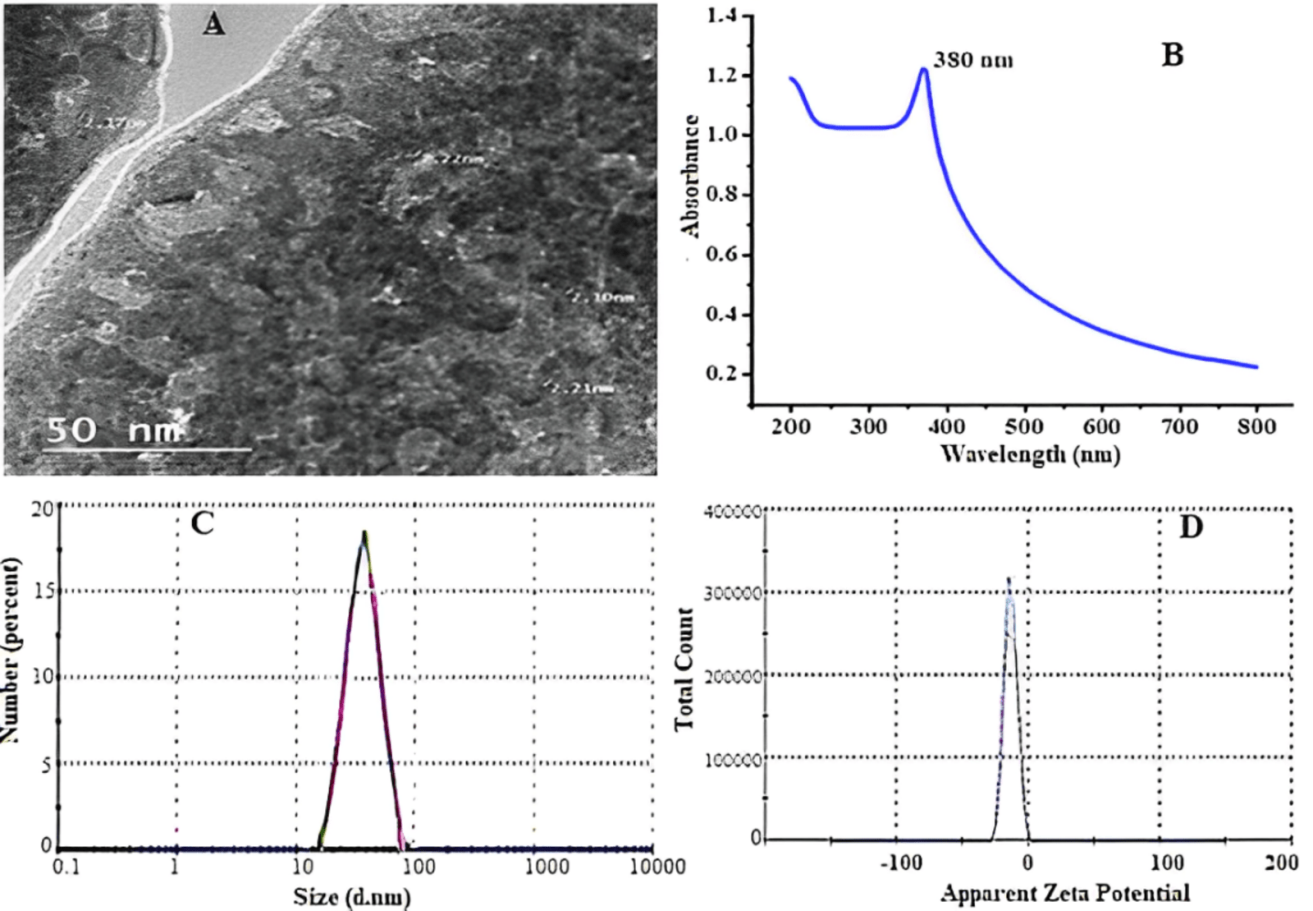
Transmission electron microscope (A), absorbance of ZnO NPs UV-VIS (B) particle size (C), and zeta potential (D) of ZnO-NPs prepared using green synthesis.

With increasing incubation time, neem leaf extracts biosynthesized ZnO-NPs, resulting in a color change from translucent to yellowish brown, indicating bio reduction of zinc oxide particles. The color shift is caused by the stimulation of surface plasmon resonance in solution. Figure 2A shows the surface morphologies and particle sizes of ZnO synthesized using neem extract by TEM. The influence of the preparation process on the photo-optical properties of zinc oxide nanoparticles was investigated using their ability to absorb UV radiation. Figure 2B depicts the UV-visible absorption spectra of zinc oxide nanoparticles. UV-visible spectral investigation yielded absorption spectra of 380 nm. In Fig. 2C, the particle size of synthesized ZnO powder is approximately 62.5 nm, and the zeta potential is shown in Fig. 2D.

### Qualitative phytochemical screening of neem leaf aqueous extract

*Azadirachta indica* has gained significance in the contemporary world setting due to its ability to provide solutions to the significant challenges confronting humanity. It is a fast-growing evergreen popular tree found commonly in India, Africa, and America. As presented in Table 1, qualitative analysis for leaf aqueous extract of *A. indica* shows the presence of terpenoids, saponins, and steroids having the highest concentration, while cardiac glycosides and tannins have moderate concentrations however alkaloids, flavonoids, and phenols have low concentrations. Therapeutic plants include a variety of secondary metabolites such as alkaloids, flavonoids, saponins, and other active compounds. These compounds have significant therapeutic properties and have been widely utilised in the medicine and pharmaceutical sectors. These secondary metabolites are documented to possess numerous biological and medicinal characteristics.

**Table 1:** Qualitative phytochemical screening of neem leaf aqueous extract.

No.	**Components**	**Abundance**
1	Alkaloids	+
2	Flavonoids	+
3	Terpenoids	+++
4	Saponins	+++
5	Steroids	+++
6	Cardiac Glycosides	++
7	Phenols	+
8	Tannins	++

**Notes.**

While: (+++) Most present; (++) Moderately present; (+) Least present.

### Total phenolic (TPC), total flavonoids (TFC) content, and antioxidant activity

Data presented in Table 2 show that the quantitative of total phenolic (TPCs by mg GAE g^−1^ dry extract), total flavonoid (TFCs by mg QE g^−1^ dry extract), and antioxidant activity (DPPH %) of neem leaf aqueous extract at different concentrations (500–1,000–1,500–2,000 µg/mL). Data declared that significant differences (*P* ≤ 0.05) were examined between the phytochemical content of *A. indica* leaves. The mean value for TPC in *A. indica* leaves was calculated as 119.00 mg GAE g^−1^ dry extract. The TFC content of *A. indica* leaves was observed as 45.67 mg QE g^−1^ dry extract. To complete the data for the aqueous leaves of *A. indica*, it is necessary to measure their antioxidant activity using DPPH. The percentage of antioxidant activity of *A. indica* leaf extract was measured using DPPH assay at various concentrations, including 500, 1,000, 1,500, and 2,000 µg/mL, and results are presented in Table 2. As the concentration of *A. indica* leaf extract increased, its antioxidant activity demonstrated a gradual increase. As the extract concentration increased from 500 to 2,000 µg/mL, the effectiveness of DPPH radical scavenging increased from 31.67% to 77.83%. The previous results reaffirmed our viewpoint regarding the antioxidant properties of plant extracts, which are attributed to the presence of polyphenolic compounds. These compounds have potential as effective antioxidant agents. Moreover, it is widely recognized that plant extracts containing phenolic and flavonoid components exhibit a substantial number of antioxidants. Phenolic compounds or polyphenols are produced by plants because of their secondary metabolism. These chemicals are frequently present in plants and have been extensively utilized due to their diverse biological actions, which include antioxidant properties.

**Table 2:** Quantitative total phenolic, total flavonoid and antioxidant activity (DPPH %) of neem leaf aqueous extract at different concentrations (500–1,000–1,500–2,000 µg/mL).

Parameters	Neem variants
TPCs (mg GAE g^−1^ dry)	119.00 ± 1.3^a^
TFs (mg QE g^−1^ dry extract)	45.67 ± 0.95^b^
Antioxidant activity (%)	% Inhibition
500	31.67 ± 0.25^d^
1,000	46.76 ± 0.87^c^
1,500	65.33 ± 1.09^b^
2,000	77.83 ± 2.11^a^

**Notes.**

Different letter in the same row indicates significant difference (*p* > 0.05).

mg GAE g-1 mg gallic acid equivalents per g dry weight mg EQ g-1 mg Quercetin equivalents per g dry weight % Inhibition Inhibition of DPPH radical

### Phenolic compounds content in neem extract by HPLC

The quantity of phenolic compounds in neem aqueous extract was found in (Table 3). The concentrations of the components that were found varied greatly. According to the standards used in the HPLC, the aqueous extract of A. indica leaves contained 19 phenolic compound fractions, which included gallic acid, chlorogenic acid, catechin, methyl gallate, coffeic acid, syringic acid, pyrocatechol, rutin, ellagic acid, coumaric acid, vanillin, ferulic acid, naringenin, daidzein, quercetin, cinnamic acid, apigenin, kaempferol, and hesperetin. In the connect, chlorogenic acid (896.76 µg/g), catechin (666.25 µg/g), gallic acid (456.68 µg/g), vanillin (423.68 µg/g), and hesperetin (372.15 µg/g) are the key five components. In addition, coumaric acid (9.75 µg/g) and cinnamic acid (8.80 µg/g) had the lowest value, while pyrocatechol and kaempferol did not detect. This variation in phenolic compounds may be responsible for this extract’s greater antioxidant effect in reducing oxidation. As a result, phenolic acids can protect against a wide range of oxidative damaged diseases.

**Table 3:** Phenolic compounds content (µg/g extract) in neem leaf aqueous extract.

N0	RT	Phenolic compound	Conc. (µg/g )
1	3.381	Gallic acid	456.68
2	4.329	Chlorogenic acid	896.76
3	4.671	Catechin	666.25
4	5.58	Methyl gallate	103.65
5	5.868	Coffeic acid	38.23
6	6.496	Syringic acid	52.06
7	6.787	Pyro catechol	0.00
8	7.954	Rutin	149.84
9	8.562	Ellagic acid	52.80
10	9.395	Coumaric acid	9.75
11	10.13	Vanillin	423.68
12	10.301	Ferulic acid	189.56
13	10.535	Naringenin	165.28
14	11.909	Daidzein	28.12
15	12.754	Querectin	41.16
16	14.082	Cinnamic acid	8.80
17	14.515	Apigenin	135.75
18	15.032	Kaempferol	0.00
19	15.673	Hesperetin	372.15

### GC-MS analysis of neem leaves ethyl acetate extract

**Figure 3:**
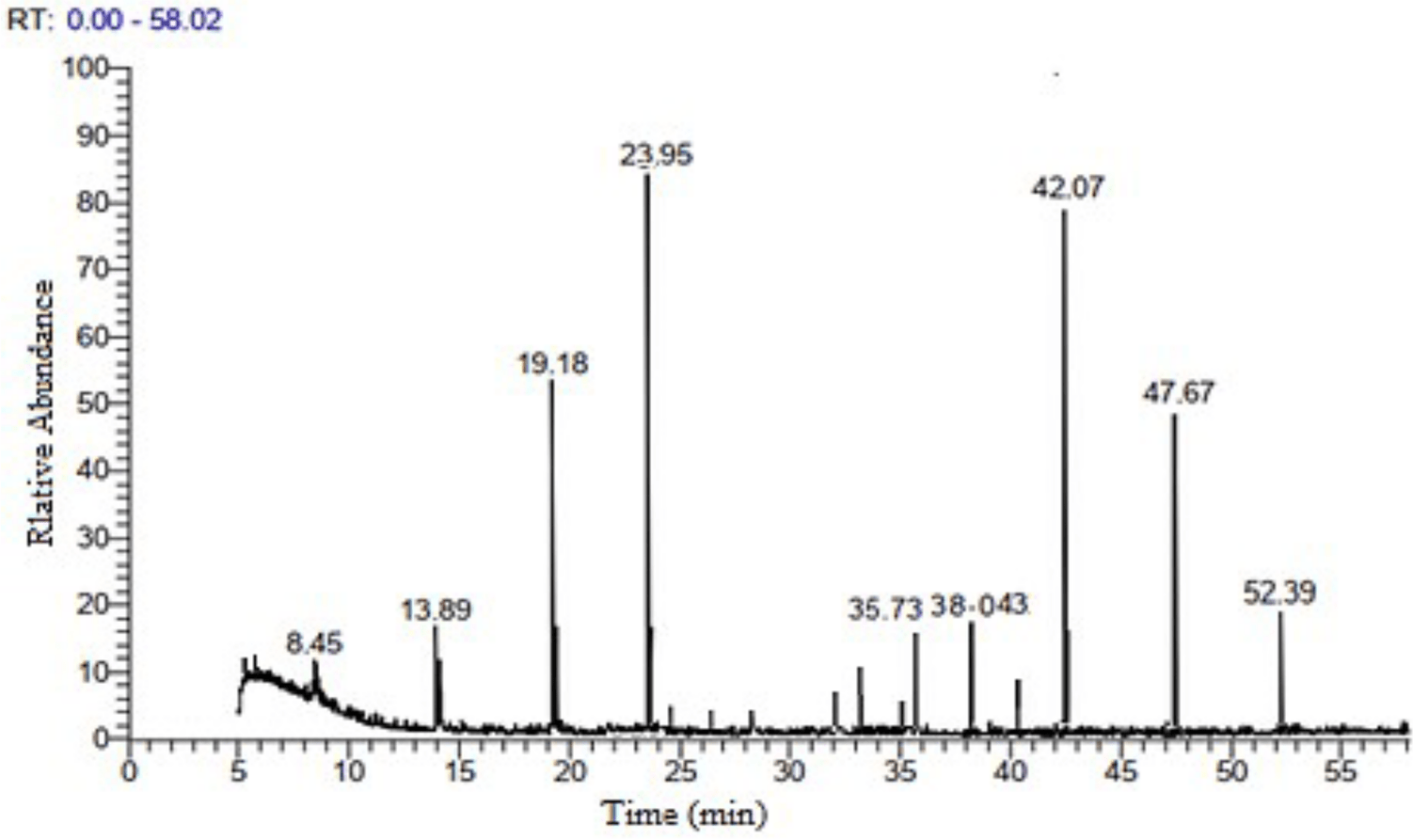
GC-MS chromatogram of neem leaves ethyl acetate extract.

The GC-MS analysis of neem leaf extract ethyl acetate fraction revealed 21 peaks, indicating the presence of 21 bioactive compounds. The chromatogram is presented in (Fig. 3, and Table 4) lists the bioactive compounds together with their retention time (RT), peak areas (%), molecular formula, and molecular weight (MW). The bioactive compounds revealed are *α*-terpinolene; citronellyl propionate; hexadecanoic acid, ethyl ester; palmitic acid, TMS derivative; phytol; distearyl phosphate; *α*-linolenic acid, TMS derivative; dicyclohexyl phthalate; heptacosane; tetracosane; pent-4-enal; Vitamin E; ethyl 2-cyano-3-Ethylpentanoate; stigmasterol; *γ*-sitosterol; *β*-sitosterol, TMS derivative; 2-diazocyclooctanone; 3-butenamide, 4-(4-chlorophenyl)-N-(1,1-dimethylethyl)-3-methyl-4-phenyl-, (Z)-; andrographolide; (2S,3R)-2,3-epoxy-5-methyl-5-hexene-1-ol and stigmasteryl tosylate. Among the twenty-one compounds identified, the major compounds present in neem ethyl acetate extract were distearyl phosphate, andrographolide, phytol, and (2S,3R)-2,3-epoxy-5-methyl-5-hexene-1-ol.

**Table 4:** GC-MS analysis of neem (*Azadirachta indica* A. Juss) leaf ethyl acetate extract.

PN	RT	Active compounds	MF	MW	Area %
1	5.18	α-Terpinolene	C_10_H_16_	136.23	0.38
2	5.22	Citronellyl propionate	C_13_H_24_O_2_	212.33	0.78
3	8.45	Hexadecanoic acid, ethyl ester	C_18_H_36_O_2_	284.47	3.12
4	13.89	Palmitic Acid, TMS derivative	C_19_H_40_O_2_Si	328.6	3.7
5	19.18	Phytol	C_20_H_40_O	296.53	9.46
6	23.95	Distearyl phosphate	C_36_H_75_O_4_P	603	29.81
7	24.11	α-Linolenic acid, TMS derivative	C_21_H_38_O_2_Si	350.61	1.00
8	26.31	Dicyclohexyl phthalate	C_20_H_26_O_4_	330.42	0.42
9	28.25	Heptacosane	C_27_H_56_	380.7	0.63
10	28.38	Tetracosane	C_24_H_50_	338.65	2.08
11	32.16	Pent-4-enal	C_5_H_8_O	84.12	0.96
12	33.21	Vitamin E	C_29_H_50_O_2_	430.71	2.72
13	35.36	Ethyl 2-Cyano-3-Ethylpentanoate	C_10_H_17_NO_2_	183.25	0.8
14	35.73	Stigmasterol	C_29_H_48_O	412.7	3.65
15	38.043	γ-Sitosterol	C_29_H_50_O	414.7	4.18
16	38.45	β-Sitosterol, TMS derivative	C_32_H_58_OSi	486.88	1.9
17	39.02	2-Diazocyclooctanone	C_8_H_12_N_2_O	152.19	1.96
18	40.76	3-Butenamide, 4-(4-chlorophenyl)-N-(1,1-dimethylethyl)-3-methyl-4-phenyl-, (Z)-	C_21_H_24_ClNO	341.88	0.89
19	42.07	Andrographolide	C_20_H_30_O_5_	350.4	18.82
20	47.67	(2S,3R)-2,3-Epoxy-5-methyl-5-hexene-1-ol	C_7_H_12_O_2_	128.17	8.53
21	52.39	Stigmasteryl tosylate	C_36_H_54_O_3_S	566.9	4.22
			Total area percent		100.01

### Cytotoxicity on A549 and HCT116

**Figure 4:**
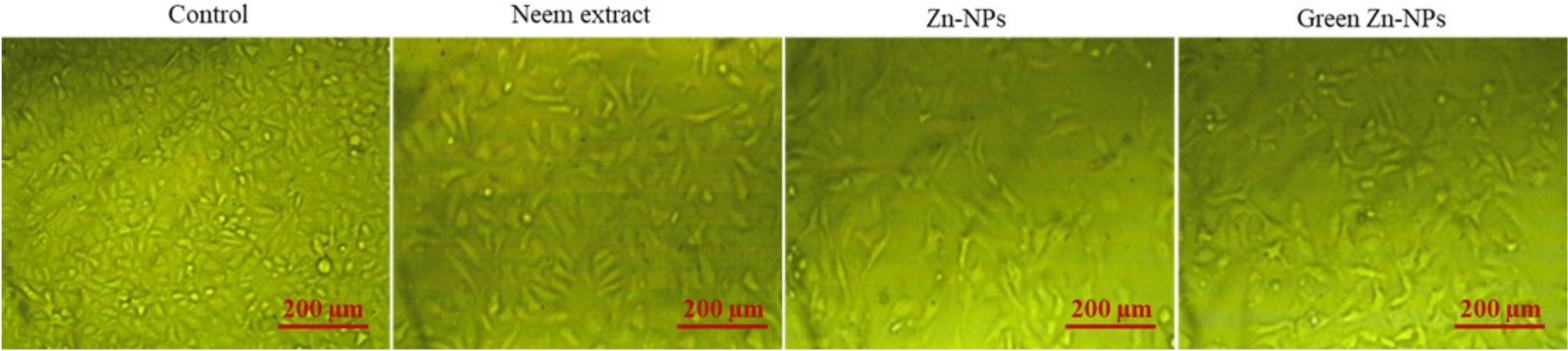
The shape of HCT 116 cells changed after being treated for 24 h with neem aqueous extract, Zn-NPs, and green synthesized ZnO-NPs at IC_50_ concentrations (350, 190, and 118 µg/mL, respectively).

**Figure 5:**
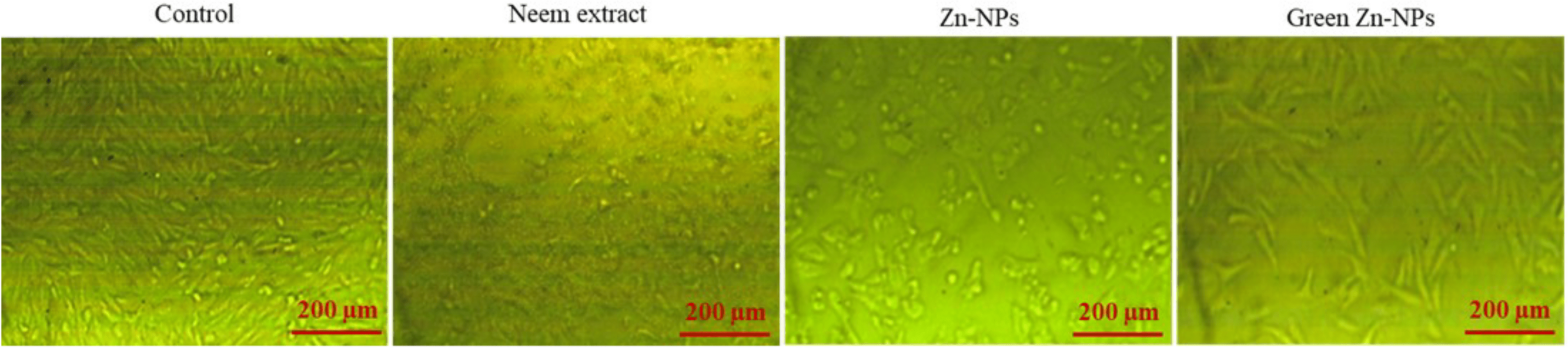
The shape of A549 cells changed after being treated for 24 h with neem aqueous extract, Zn-NPs, and green synthesized ZnO-NPs at IC_50_ concentrations (227, 197, and 111 µg/mL, respectively).

To explore the possible cytotoxic effects of neem aqueous extract, ZnO-NPs, and green synthesized ZnO-NPs on colorectal (HCT116) and lung (A549) cancer cells, different concentrations of the extract and nanoparticles were used to treat the cells, and the resulting antiproliferative effects were measured using MTT assay. Following the administration of neem aqueous extract, ZnO-NPs, and green synthesized ZnO-NPs, the morphology of A549 and HCT116 cell lines exhibited aberrations in comparison to the untreated cell control. The application of neem aqueous extract, ZnO-NPs, and green-synthesized ZnO-NPs resulted in observable morphological alterations in the treated cells, including cell shrinkage and suspension in the media (Figs. 4 and 5).

### MTT-assay

**Figure 6:**
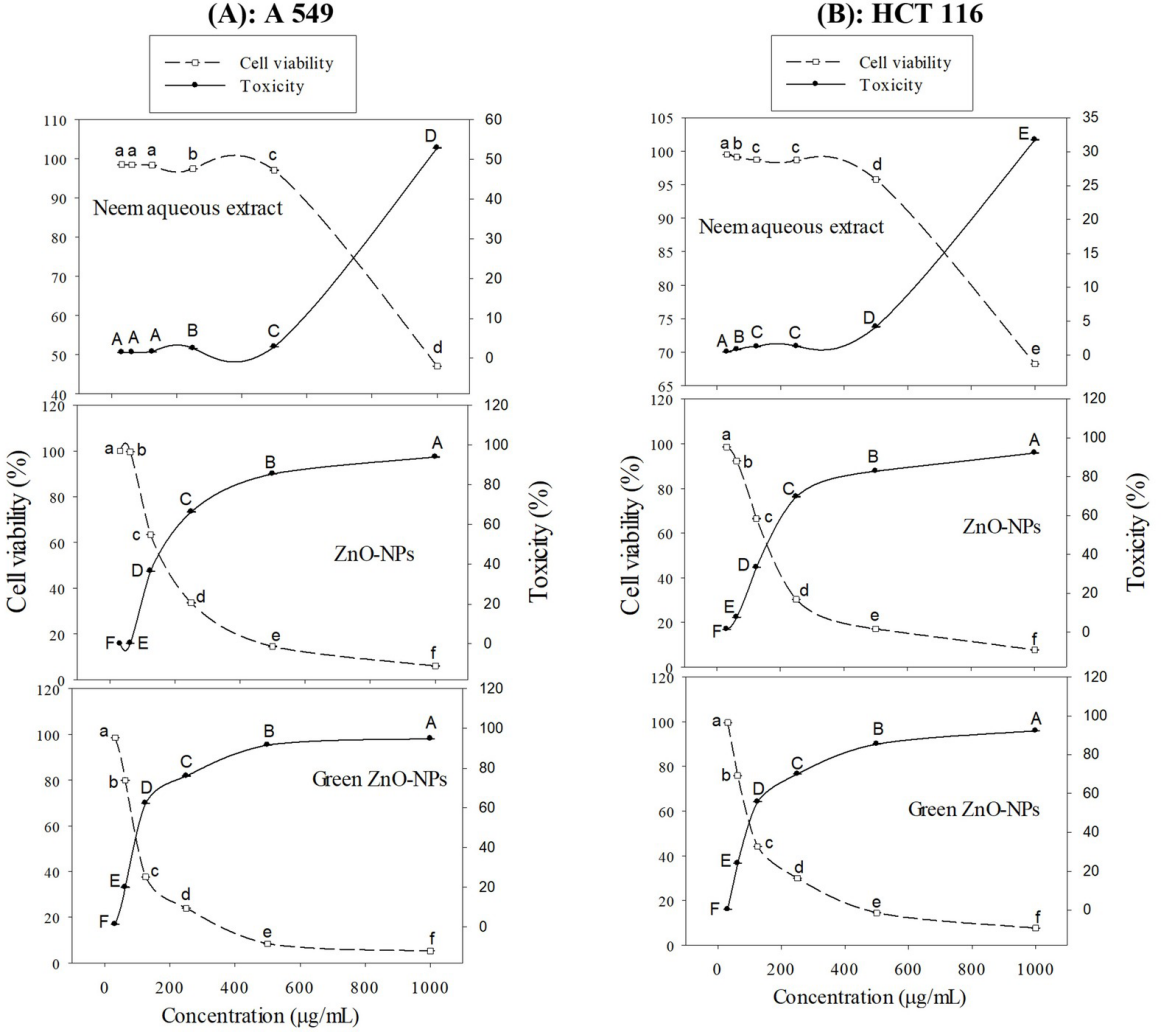
Cell viability (%) and Toxicity (%) of A549 (A), and HCT 116 (B) cell lines treated with ZnO-NPs, neem aqueous extract, and green synthesized ZnO-NPs at different concentrations. Different letters indicate significant differences among the cell viability (small letters) and toxicity (capital letters) according to Tukey’s HSD test (*p* ≤ 0.05).

**Figure 7:**
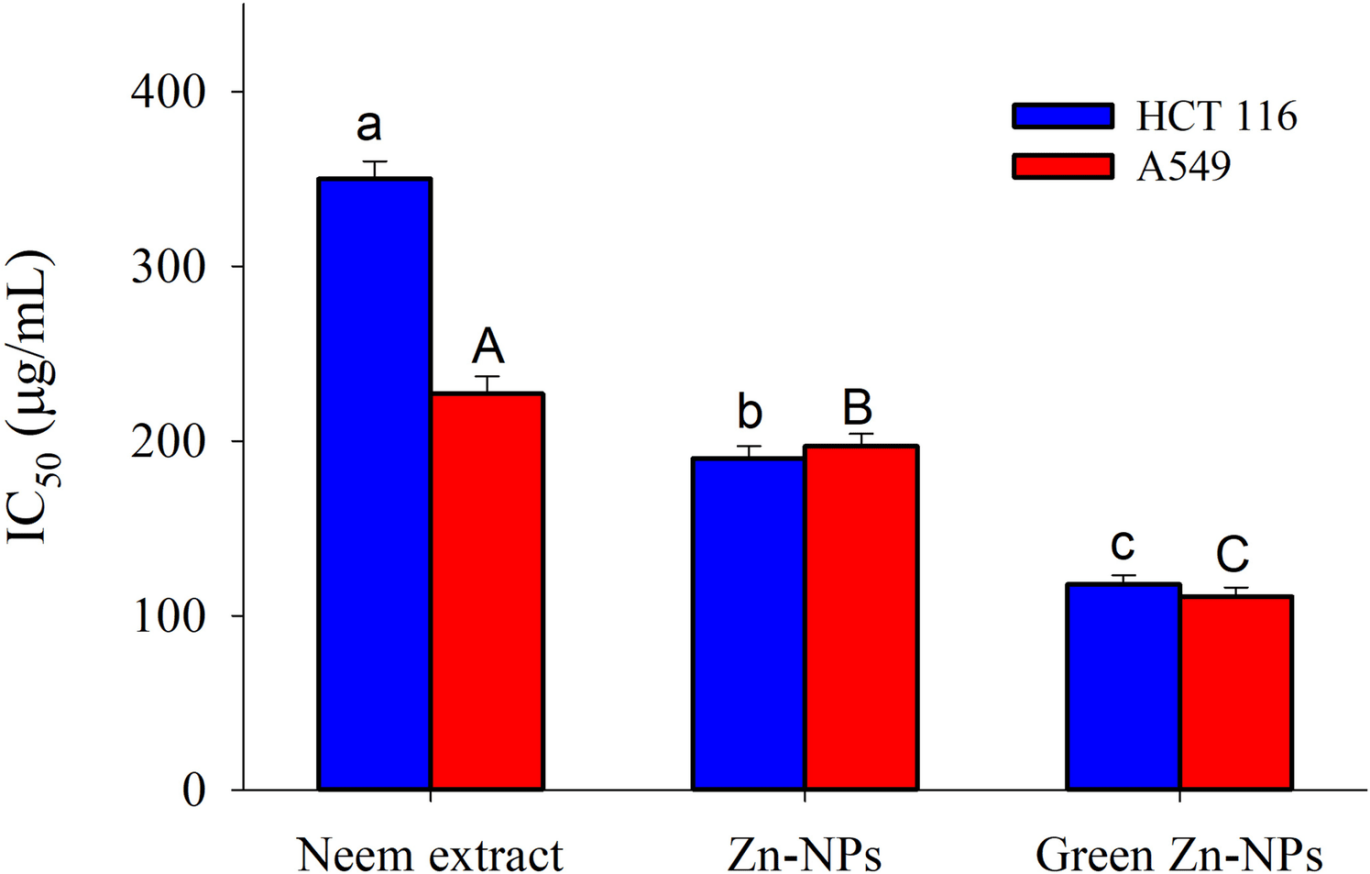
The 50% inhibitory concentration (IC_50_) values of ZnO-NPs, neem extract, and green synthesized ZnO-NPs for A549 and HCT 116 cell lines after 24 hours of treatment. The different letters indicate significant differences among the IC50 against HCT 116 (small letters) and the IC50 against A549 (capital letters), according to Tukey’s HSD test (*p* ≤ 0.05).

The toxicity (%) and cell viability (%) of the A549 and HCT116 cell lines, when subjected to treatment with ZnO-NPs, neem aqueous extract, and green synthesized ZnO-NPs at different concentrations, are depicted in Figs. 6A and 6B. The linear correlation between cell viability and toxicity from the tested substances is presented in Figs. 4 & 5. The data clearly demonstrates a negative correlation between the concentration of the tested materials and the overall cell viability percentage. The MTT assay demonstrated that the tested materials exhibited a concentration-dependent inhibitory effect on the growth of human cancer cell lines (A549 and HCT116). The findings demonstrate that both the extract and nanoparticles exhibited a dose-dependent inhibition of cell proliferation, as illustrated in Figs. 4, 5, 6A, 6B and 7. Our study revealed that the antiproliferative effect of green synthesized Zn-NPs was much superior to that of ZnO-NPs and neem aqueous extract. This suggests that the cytotoxic activities of neem aqueous extract and ZnO-NPs were enhanced upon their conjugation. Figures 6A and 6B demonstrate that ZnO-NPs can reduce the cell viability percentage of A549 and HCT16 cell lines.

The 50% inhibitory concentration (IC_50_) values of ZnO-NPs, neem extract, and green synthesized ZnO-NPs for A549 and HCT116 cell lines after 24 h of treatment are presented in Fig. 7. The neem aqueous extract had the lowest IC_50_ against A 549 (227 µg/mL), with HCT16 coming in second (350 µg/mL). On the other hand, Zn-NPs had the lowest IC_50_ against HCT116 (190 µg/mL), with A 549 coming in second (197 µg/mL). Finally, the IC_50_ value for green Zn-NPs was found to be 111 µg/mL for A 549 and 118 µg/mL for HCT116.

**Figure 8:**
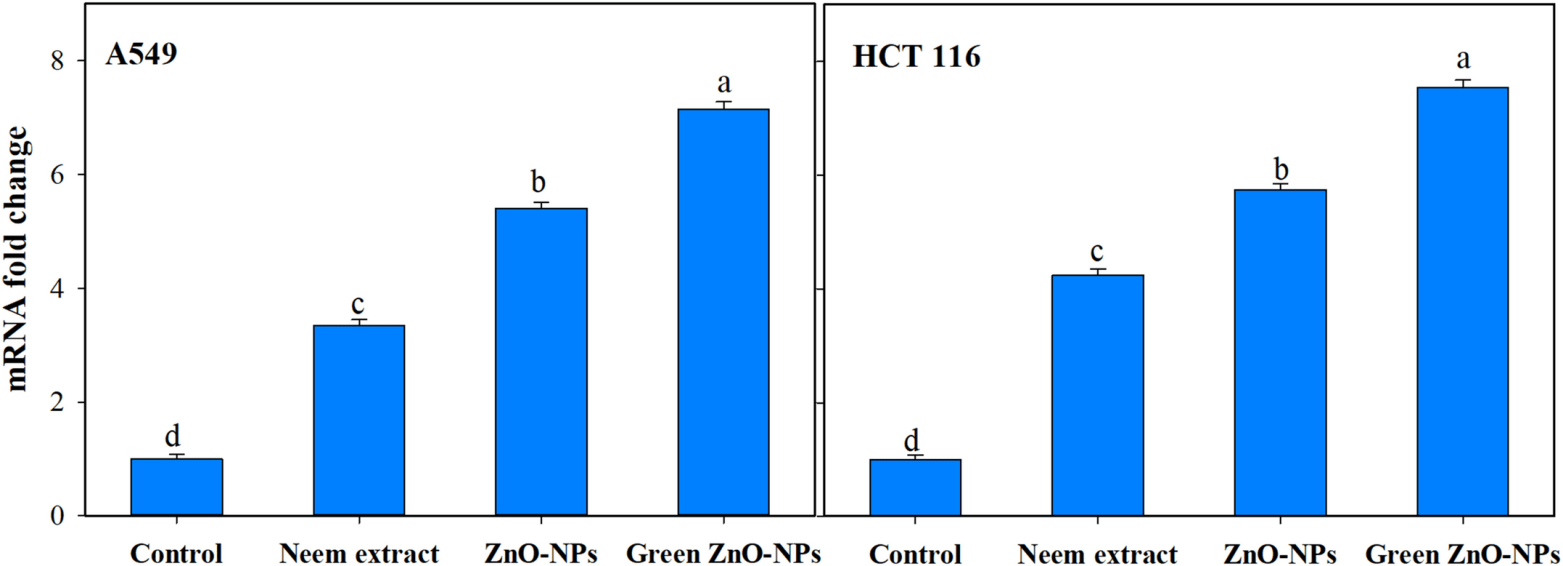
Effect of neem aqueous extract, ZnO-NPs, and green synthesized ZnO-NPs on caspase 9 gene expression of two human cancer cell lines (A549, and HCT 116). Cells were treated with the concentration causing the IC_50_ for each cell line for 24 h and their mRNA levels were evaluated by quantitative real-time PCR. The different letters indicate significant differences among treatments, according to Tukey’s HSD test (*p* ≤ 0.05).

The utilization of green synthesis techniques in the production of ZnO-NPs has been found to have a notable impact on the upregulation of caspase-9 transcript expression (Fig. 8). This effect is observed to be much greater when compared to other treatments involving neem extract and Zn-NPs, as well as the control group consisting of untreated cells. Treating A549 and HCT116 cells with 111 and 118 µg/mL Zn-NPs increased caspase-9 expression by 7.14 and 7.53-fold, respectively, after 24 h compared to untreated controls.

### Antibacterial activity

### MIC estimation

Different concentrations of neem extract, ZnO-NPs, and green synthesized ZnO-NPs (0, 0.5, 1, 2, 5, 10, and 20 µg/mL) were tested for antibacterial activity. The results showed that the MIC of neem aqueous extract, ZnO-NPs, and green synthesized ZnO-NPs against Gram-positive *S. aureus* and *L. monocytogenes* were 10, 5, and 1 µg/mL, respectively (Table 5). The MIC against Gram-negative bacteria *S. Enteritidis* and *E. coli* was 20, 10, and 5 µg/mL, respectively (Table 5).

### TEM

**Figure 9:**
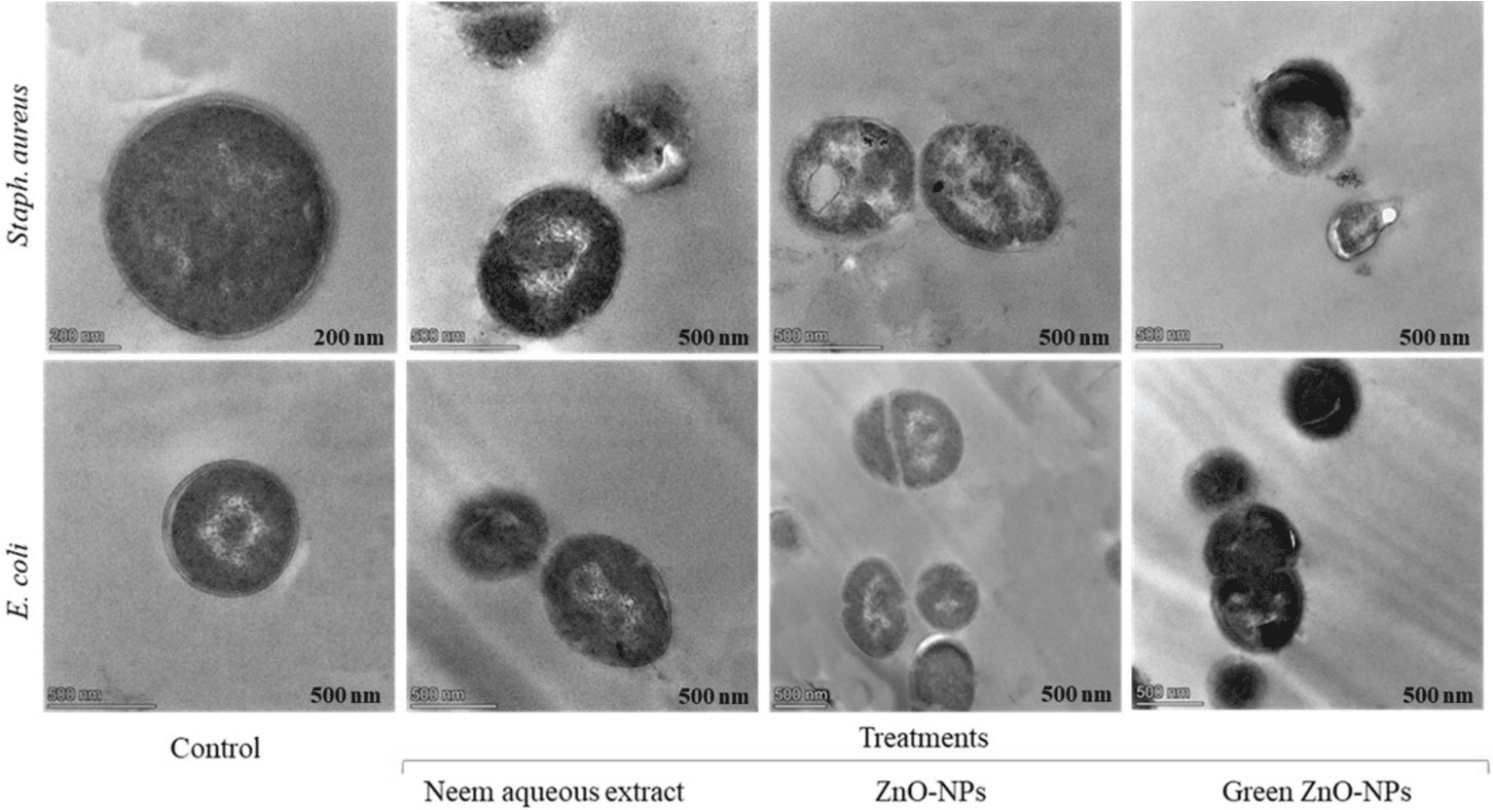
Transmission electron microscopy graphs *Staph. aureus*, and *E. coli* as subjected to 1 MIC of neem aqueous extract, ZnO-NPs, and green synthesized ZnO-NPs.

Following the addition of neem aqueous extract, ZnO-NPs, and green-synthesized ZnO-NPs (at a concentration of 1 MIC) to MHB media containing *Staph. aureus* and *E. coli*, TEM pictures revealed a reduction in the proportion of intact cells after 4 h of incubation at 37 °C (Fig. 9).

**Table 5:** Minimum inhibitory concentration (MIC) of neem aqueous extract, ZnO-NPs, and green synthesized ZnO-NPs against gram positive (*L. monocytogenes* and *Staph. Aureus*) and gram negative (*S. Enteritidis* and *E. coli*) bacteria.

**Bacterial strains**	**MIC (µg/mL)**		
	**Neem extract**	**ZnO-NPs**	**Green ZnO-NPs**
Gram positive			
*S. aureus*	10	5	1
*L. monocytogenes*	10	5	1
Gram negative			
*E. coli*	20	10	5
*S. Enteritidis*	20	10	5

The TEM pictures showed that bacterial cells that had survived displayed diverse deformations. The compounds subjected to testing (1 MIC) exhibited comparable impacts on *Staph. aureus*, including cellular contraction, wrinkling of the cell membrane, creation of pores, and depletion of viable cellular matter. Comparable findings were likewise noted for *E. coli*.

## Discussion

**Figure 10:**
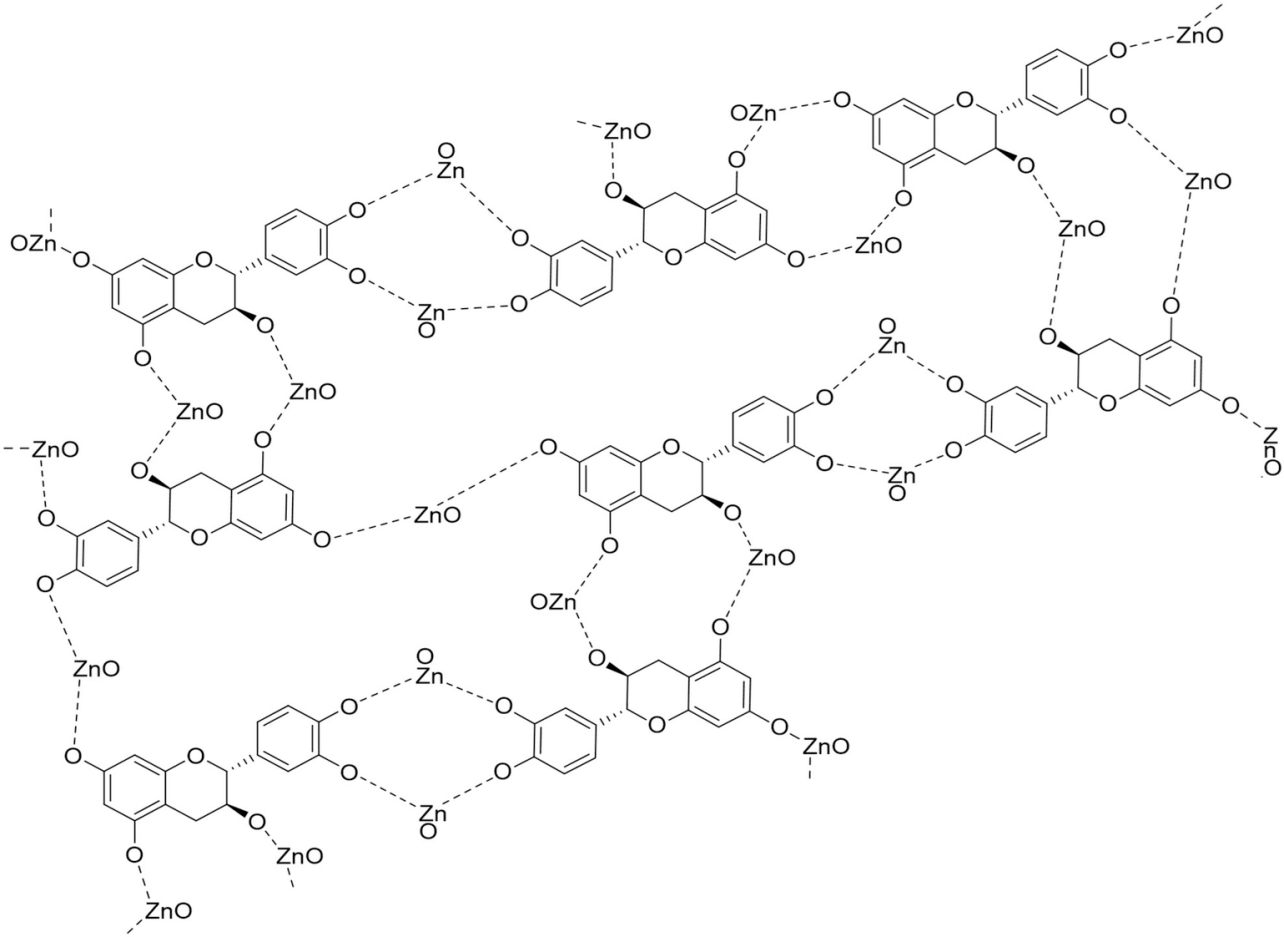
The plausible mechanism of formation of zinc oxide nanoparticles (ZnO-NPs) from neem aqueous leaf extract.

Nanoparticles can be synthesized using a variety of methods; nonetheless, environmentally friendly methods have become chosen over traditional chemical and physical procedures. The synthesis of nanoparticles by chemical and physical methods can be expensive, time-consuming, and energy-intensive, have a detrimental influence on the environment, and leave toxic compounds on the surface. These nanoparticles cannot be employed in medicinal applications (El-Belely et al., 2021). This study used a biological technique to synthesize ZnO-NPs. *Azadirachta indica* leaves aqueous extract was utilized as a reducing and stabilizing agent to synthesize ZnO-NPs. Plant-based synthesis methods have several benefits, such as being easy to deal with, affordable, and possible without the use of chemical solvents or harmful chemicals (Sohail et al., 2020; Fouda, 2023). The phytochemical analysis of neem extract indicates that it consists of several distinct components (Gupta et al., 2017). This plant group is particularly rich in phenolic chemicals, which are employed as a reducing agent in the synthesis of ZnO-NPs. Although the exact mechanism for forming ZnO-NPs from plant extracts is unknown, it is thought that polar groups play a role (Jha & Prasad, 2010; Anandan et al., 2019). Hence, one of the plausible mechanisms for the reducing and capping effects of the plant extract during the formation of ZnO-NPs is represented in Fig. 10. According to the reduction of the zinc nitrate using *Azadirachta indica* extract as a green synthesis of ZnO-NPs, the compounds containing (–OH, -NH, and NH_2_) can reduce (Zn^2+^) ion. They exhibit appropriate reducing effects in addition to high stabilizing properties during ZnONPs preparation (Anandan et al., 2019; Alharbi, Abaker & Makawi, 2023; Eltak et al., 2023). The chemical and green synthesis of ZnO-NPs has been extensively explored and confirmed using TEM, UV-Vis spectrophotometer, and DLS analysis. . These results are similar to some results of studies that used neem extract to prepare zinc oxide nanoparticles, but with different sizes. In this regard, the sizes formed of ZnO-NPs were 9.6 to 25.5 nm (Bhuyan et al., 2015), and 19.57 ± 1.56 nm (Sohail et al., 2020).

The analysis of phytochemicals in the neem aqueous extract: phenolics, flavonoids, alkaloids, tannins, terpenoids, saponins, steroids, and cardiac glycosides were similar to earlier findings (Dash, Dixit & Sahoo, 2017). Plant secondary metabolites, known as phytoconstituents, are a useful and unique source of food supplements and medications. Extensive research has thoroughly established their numerous functions and uses (García-Cruz, Salinas-Moreno & Valle-Guadarrama, 2012). In the present study, some of the main phenolic and flavonoids found in the extract by HPLC analysis are hesperetin, naringenin, cinnamic acid, apigenin, kaempferol, naringenin, coumaric acid, vanillin, ferulic acid, naringenin, daidzein, quercetin, cinnamic acid, apigenin, kaempferol, and apigenin. These compounds have antioxidant (Sani & Baburo, 2020; Nagano & Batalini, 2021), antibacterial (Elizabeth Babatunde et al., 2019), and anticancer (Azhagu Madhavan, 2021; Madhavan, 2021) properties. The inquiry used GC-MS to analyze the ethyl acetate fraction of neem leaf extract. The research findings revealed the presence of 21 bioactive compounds. Although 21 compounds occurred, only 14 had a high area percent (peaks), with the remaining 7 having a low area percent, as seen in Fig. 3. The neem ethyl acetate extract contains hydrocarbons, terpenoids, phenolics, alkaloids, fatty acids, and their derivatives. The literature study indicates that most of the prevalent compounds in neem have biological activity (Cock et al., 2009; Lucantoni et al., 2010; Hossain & Nagooru, 2011). Phenols and flavonoids have an aromatic ring with at least one hydroxyl group substituted. It forms chelate compounds with metal ions. Consequently, they are prone to oxidation. Therefore, they serve as great entities for donating electrons (Sankhalkar, 2014).

To assess the anticancer activity of green and chemically synthesized ZnONPs compared to neem leaf aqueous extract, the MTT assay was used. Based on the results of this study, the eco-friendly ZnO-NPs had the biggest effect, as shown by the lowest IC_50_ value. They were followed by ZnO-NPs and then the neem aqueous extract. The improved surface chemistry of ZnO-NPs synthesized using green methods and ZnO-NPs, when compared to neem aqueous extract, can be linked to their reduced size. The variation in size may enhance their contact with cancer cells (Mthana et al., 2022). While numerous research has documented the anticancer properties of ZnO-NPs, the precise underlying mechanism remains to be fully understood. ZnO-NPs have been shown to help fight cancer. This is likely because they create reactive oxygen species (ROS) on their surface. Furthermore, the breaking down of the particles and the subsequent release of free Zn^2+^ ions help cells make ROS (Kasemets et al., 2009; Wang et al., 2014; El-Beltagi et al., 2022). Several studies have shown that ZnO NPs can kill cells by creating ROS (Sirelkhatim et al., 2015a). There is evidence from several studies that ROS cause lipid peroxidation, enzyme inactivation, and membrane degradation. The main reasons why ZnO NPs are thought to fight cancer are these mechanisms (Yu et al., 2013; Wang et al., 2015). To find alternatives that are better for the environment, scientists are looking into long-term ways to make metal nanoparticles and metal oxide nanoparticles using plant extracts. The approaches are characterized by their relative ease, cost-effectiveness, and environmental superiority as compared to chemical and physical alternatives (Alshameri & Owais, 2022). The neem water extract utilized in this investigation contained various bioactive compounds, including phenolics, flavonoids, alkaloids, tannins, terpenoids, saponins, steroids, and cardiac glycosides. Flavonoids, terpenoids, polyphenols, alkaloids, phenolic acids, and other secondary metabolites have been identified as compounds that play a crucial role in the reduction of metal ions to zerovalent metals or in the stabilization of metal nanoparticles (MNPs). Isoflavonoids, flavonols, flavones, and flavanones represent a range of chemical classes. The capacity of flavonoids to form coordination complexes with metal ions is attributed to the presence of hydroxyl and carbonyl functional groups. The manufacture of metal oxide nanoparticles (NPs) can employ various amino acids, sugars, or fatty acids as readily accessible reducing and capping agents (Sahu et al., 2016). The findings of the study demonstrated that the ZnO-NPs, which were synthesized using a green method, exerted a significant impact on the viability of A549 and HCT 116 cells. Notably, this effect was observed even at the lowest doses of the samples administered. The results shown in this study exhibit a strong correlation with the conclusions reported by Suresh et al. (2018), Umamaheswari et al. (2021), Naser et al. (2021), and Selim et al. (2020).

In this study, the antibacterial activity of neem extract, ZnO-NPs, and green synthesized ZnO-NPs was evaluated against both gram-positive and gram-negative bacteria. The highest activity was recorded for green synthesized ZnO-NPs then ZnO-NPs, and finally neem extract. The present study recorded the phytochemical components of *A. indica*, including saponins, steroids, terpenes, tannins, glycosides, alkaloids, flavonoids, and phenols. These phytochemicals may account for the antibacterial activity of neem. Numerous studies have established a correlation between the existence of bioactive chemicals in plant materials and their antibacterial properties (El-Mahmood, Ogbonna & Raji, 2010; Francine, Jeannette & Pierre, 2015; Itelima et al., 2016). The antibacterial properties of ZnO-NPs can be ascribed to their direct contact with cellular walls, resulting in membrane distortion and rupture (Brayner et al., 2006; Zhang et al., 2007; Sarwar et al., 2016). The ability of ZnO-NPs to kill *Vibrio cholerae* bacteria and how they cause harm was studied (Sarwar et al., 2016). ZnO NPs interacted with *Vibrio cholerae* and made the membranes more fluid and depolarized. This led to protein leakage and changes in the structure of the *Vibrio cholerae* cells. It has been seen that bacteria treated with ZnO-NPs often have cell membrane damage, which is very toxic. The impact of ZnO-NPs on *E. coli* was investigated by Leung et al. (2016), who observed that greater concentrations of ZnO-NPs resulted in the identification of cell damage sites. The damage seen in the cell membrane is caused by ZnO-NPs interacting with bacterial membranes. There are bad effects on the molecular structure of phospholipids when certain factors interact with each other, which damages the cell membrane.

## Conclusions

The study found that green ZnO-NPs with non-spherical shapes, synthesized using neem aqueous extracts, had strong antibacterial activity against various Gram-positive and Gram-negative bacteria. In addition, the ZnO-NPs exhibited significant anticancer effects on HCT116 and A549 cancer cells. ZnO-NPs exhibit strong antibacterial and anticancer properties, making them promising candidates for novel therapeutic applications in biomedical fields.

## Supplemental Information

10.7717/peerj.17588/supp-1 Supplemental Information 1 Cell line toxicity raw data

10.7717/peerj.17588/supp-2 Supplemental Information 2 Author justification

## Additional Information and Declarations

### Data Availability

The following information was supplied regarding data availability: The raw data is available in the Supplemental File.

## References

[ref-1] Abd Elhamid MA, Mandour AES, Ismail TA, Al-Zohairy AM, Almowallad S, Alqahtani LS, Osman A (2022). Powerful antioxidants and cytotoxic activities of the methanol extracts from eight soybean cultivars. Molecules.

[ref-2] Abdel-Hamid M, Osman A, El-Hadary A, Romeih E, Sitohy M, Li L (2020a). Hepatoprotective action of papain-hydrolyzed buffalo milk protein on carbon tetrachloride oxidative stressed albino rats. Journal of Dairy Science.

[ref-3] Abdel-Hamid M, Otte J, De Gobba C, Osman A, Hamad E (2017). Angiotensin I-converting enzyme inhibitory activity and antioxidant capacity of bioactive peptides derived from enzymatic hydrolysis of buffalo milk proteins. International Dairy Journal.

[ref-4] Abdel-Hamid M, Romeih E, Saporito P, Osman A, Mateiu RV, Mojsoska B, Jenssen H (2020b). Camel milk whey hydrolysate inhibits growth and biofilm formation of Pseudomonas aeruginosa PAO1 and methicillin-resistant Staphylococcus aureus. Food Control.

[ref-5] Abdel-Rahim EA, El-Beltagi HS (2010). Constituents of apple, parsley and lentil edible plants and their therapy treatments for blood picture as well as liver and kidney functions against lipidemic disease. Electronic Journal of Environmental, Agricultural and Food Chemistry.

[ref-6] Abdel Rahman AN, Amer SA, Behairy A, Younis EM, Abdelwarith AA, Osman A, Moustafa AA, Davies SJ, Ibrahim RE (2023). Using *Azadirachta indica* protein hydrolysate as a plant protein in Nile tilapia (*Oreochromis niloticus*) diet: effects on the growth, economic efficiency, antioxidant-immune response and resistance to Streptococcus agalactiae. Journal of Animal Physiology and Animal Nutrition.

[ref-7] Abdel-Shafi S, Al-Mohammadi A-R, Sitohy M, Mosa B, Ismaiel A, Enan G, Osman A (2019a). Antimicrobial activity and chemical constitution of the crude, phenolic-rich extracts of Hibiscus sabdariffa, Brassica oleracea and Beta vulgaris. Molecules.

[ref-8] Abdel-Shafi S, El-Nemr M, Enan G, Osman A, Sitohy B, Sitohy M (2022). Isolation and characterization of antibacterial conglutinins from lupine seeds. Molecules.

[ref-9] Abdel-Shafi S, Osman A, Al-Mohammadi A-R, Enan G, Kamal N, Sitohy M (2019b). Biochemical, biological characteristics and antibacterial activity of glycoprotein extracted from the epidermal mucus of African catfish (Clarias gariepinus). International Journal of Biological Macromolecules.

[ref-10] Abdel-Shafi S, Osman A, Enan G, El-Nemer M, Sitohy M (2016). Antibacterial activity of methylated egg white proteins against pathogenic G+ and G- bacteria matching antibiotics. SpringerPlus.

[ref-11] Afify AEMM, El-Beltagi HS (2011). Effect of insecticide cyanophos on liver function in adult male rats. Fresenius Environmental Bulletin.

[ref-12] Ahmed S, Ahmad M, Swami BL, Ikram S (2016). Green synthesis of silver nanoparticles using *Azadirachta indica* aqueous leaf extract. Journal of Radiation Research and Applied Sciences.

[ref-13] Ahamed AJ, Kumar PV (2016). Synthesis and characterization of ZnO nanoparticles by co-precipitation method at room temperature. Journal of Chemical and Pharmaceutical Research.

[ref-14] Alharbi FN, Abaker ZM, Makawi SZA (2023). Phytochemical substances—mediated synthesis of zinc oxide nanoparticles (ZnO NPS). Inorganics.

[ref-15] Al-Mohammadi A-R, Osman A, Enan G, Abdel-Shafi S, El-Nemer M, Sitohy M, Taha M (2020). Powerful antibacterial peptides from egg albumin hydrolysates. Antibiotics.

[ref-16] Al Saiqali M, Tangutur AD, Banoth C, Bhukya B (2018). Antimicrobial and anticancer potential of low molecular weight polypeptides extracted and characterized from leaves of *Azadirachta indica*. International Journal of Biological Macromolecules.

[ref-17] Alshameri AW, Owais M (2022). Antibacterial and cytotoxic potency of the plant-mediated synthesis of metallic nanoparticles Ag NPs and ZnO NPs: a review. OpenNano.

[ref-18] Amer SA, Al-Khalaifah HS, Gouda A, Osman A, Goda NI, Mohammed HA, Darwish MI, Hassan AM, Mohamed SK (2022a). Potential effects of anthocyanin-rich roselle (*Hibiscus sabdariffa* L.) extract on the growth, intestinal histomorphology, blood biochemical parameters, and the immune status of broiler chickens. Antioxidants.

[ref-19] Amer SA, El-Araby DA, Tartor H, Farahat M, Goda NIA, Farag MFM, Fahmy EM, Hassan AM, Abo El-Maati MF, Osman A (2022b). Long-term feeding with curcumin affects the growth, antioxidant capacity, immune status, tissue histoarchitecture, immune expression of proinflammatory cytokines, and apoptosis indicators in Nile Tilapia, *Oreochromis niloticus*. Antioxidants.

[ref-20] Anandan S, Mahadevamurthy M, Ansari MA, Alzohairy MA, Alomary MN, Farha Siraj S, Nagaraja SH, Chikkamadaiah M, Ramachandrappa LT, Krishnappa HKN, Ledesma AE, Nagaraj AK, Urooj A (2019). Biosynthesized ZnO-NPs from Morus indica attenuates methylglyoxal-induced protein glycation and RBC damage: *in-vitro, in-vivo* and molecular docking study. Biomolecules.

[ref-21] Asadi M, Shanehbandi D, Kermani TA, Sanaat Z, Zafari V, Hashemzadeh S (2018). Expression level of caspase genes in colorectal cancer. Asian Pacific Journal of Cancer Prevention.

[ref-22] Azhagu Madhavan S (2021). Phytochemical analysis and anticancer activity of *Azadirachta Indica* ethanolic extract against A549 human lung cancer cell line. Journal ISSN.

[ref-23] Balamurugan V, Balakrishnan V, Sundaresan A (2015). GC-MS analysis of leaf and bark extract of *Moringa concanensis* Nimmo, a Siddha medicinal plant of South India. European Journal of Biotechnology and Bioscience.

[ref-24] Bellik Y, Boukraâ L, Alzahrani HA, Bakhotmah BA, Abdellah F, Hammoudi SM, Iguer-Ouada M (2012). Molecular mechanism underlying anti-inflammatory and anti-allergic activities of phytochemicals: an update. Molecules.

[ref-25] Bhuyan T, Mishra K, Khanuja M, Prasad R, Varma R (2015). Biosynthesis of zinc oxide nanoparticles from *Azadirachta indica* for antibacterial and photocatalytic applications. Materials Science in Semiconductor Processing.

[ref-26] Brayner R, Ferrari-Iliou R, Brivois N, Djediat S, Benedetti MF, Fiévet FJ (2006). Toxicological impact studies based on *Escherichia coli* bacteria in ultrafine ZnO nanoparticles colloidal medium. Nano Letters.

[ref-27] Chandrasekaran S, Anusuya S, Anbazhagan V (2022). Anticancer, anti-diabetic, antimicrobial activity of zinc oxide nanoparticles: a comparative analysis. Journal of Molecular Structure.

[ref-28] Cock I, Setzer W, Ruebhart K, El Dahshan O, Tomczyk M (2009). An anti-diabetic and hypolipidemic effects from *Azadirachta indica* leaves. African Journal of Biotechnology.

[ref-29] Dash SP, Dixit S, Sahoo S (2017). Phytochemical and biochemical characterizations from leaf extracts from *Azadirachta Indica*: an important medicinal plant. Biochemistry and Analytical Biochemistry.

[ref-30] Duangjai A, Nuengchamnong N, Lee L-H, Goh B-H, Saokaew S, Suphrom N (2019). Characterisation of an extract and fractions of *Azadirachta indica* flower on cholesterol lowering property and intestinal motility. Natural Product Research.

[ref-31] Dytham C (2011). Choosing and using statistics: a biologist’s guide.

[ref-32] Ebrahim AE, Abd El-Aziz NK, Elariny EY, Shindia A, Osman A, Hozzein WN, Alkhalifah DHM, El-Hossary DJ (2022). Antibacterial activity of bioactive compounds extracted from red kidney bean (*Phaseolus vulgaris* L.) seeds against multidrug-resistant Enterobacterales. Frontiers in Microbiology.

[ref-33] El-Belely EF, Farag MM, Said HA, Amin AS, Azab E, Gobouri AA, Fouda A (2021). Green synthesis of zinc oxide nanoparticles (ZnO-NPs) using Arthrospira platensis (Class: Cyanophyceae) and evaluation of their biomedical activities. Nanomaterials.

[ref-34] El-Beltagi HS, Dhawi F, Ashoush IS, Ramadan KMA (2020). Antioxidant, anti-cancer and ameliorative activities o, f Spirulina platensis and pomegranate juice against hepatic damage induced by CCl4. Notulae Botanicae Horti Agrobotanici Cluj-Napoca.

[ref-35] El-Beltagi HS, El-Mahdy OM, Mohamed HI, El-Ansary AE (2022). Antioxidants, antimicrobial, and anticancer activities of purified chitinase of talaromyces funiculosus strain CBS 129594 biosynthesized using crustacean bio-wastes. Agronomy.

[ref-36] El-Beltagi HS, Mohamed HI, Abdelazeem AS, Youssef R, Safwat G (2019). GC-MS analysis, antioxidant, antimicrobial and anticancer activities of extracts from Ficus sycomorus fruits and leaves. Notulae Botanicae Horti Agrobotanici.

[ref-37] El-Mahmood A, Ogbonna O, Raji M (2010). The antibacterial activity of Azadarichta indica (neem) seeds extracts against bacterial pathogens associated with eye and ear infections. Journal of Medicinal Plants Research.

[ref-38] El-Saber M (2021). Effect of biosynthesized Zn and Se nanoparticles on the productivity and active constituents of garlic subjected to saline stress. Egyptian Journal of Desert Research.

[ref-39] Elizabeth Babatunde D, Otusemade GO, Elizabeth Ojewumi M, Agboola O, Oyeniyi E, Deborah Akinlabu K (2019). Antimicrobial activity and phytochemical screening of neem leaves and lemon grass essential oil extracts. International Journal of Mechanical Engineering and Technology.

[ref-40] Elshafie HS, Osman A, El-Saber MM, Camele I, Abbas E (2023). Antifungal activity of green and chemically synthesized ZnO nanoparticles against alternaria citri, the causal agent citrus black rot. The Plant Pathology Journal.

[ref-41] Eltak NA, Gniedy NA, Abdel-Haleem DR, Farag SM (2023). Based on GC-MS analysis: an evaluation activity of some algal extracts against Culex pipiens L. (Diptera: Culicidae). Egyptian Journal of Aquatic Biology & Fisheries.

[ref-42] Elumalai P, Gunadharini DN, Senthilkumar K, Banudevi S, Arunkumar R, Benson CS, Sharmila G, Arunakaran J (2012). Ethanolic neem (*Azadirachta indica* A. Juss) leaf extract induces apoptosis and inhibits the IGF signaling pathway in breast cancer cell lines. Biomedicine & Preventive Nutrition.

[ref-43] Enan GA, Abdel-Shafi S, El-Nemr M, Shehab W, Osman A, Sitohy M, Sitohy B (2023). Controlling bacterial biofilm formation by native and methylated lupine 11S globulins. Frontiers in Microbiology.

[ref-44] Fekry M, Yahya G, Osman A, Al-Rabia MW, Mostafa I, Abbas H (2022). GC-MS analysis and microbiological evaluation of caraway essential oil as a virulence attenuating agent against Pseudomonas aeruginosa. Molecules.

[ref-45] Fouda F (2023). Synthesis and characterization of zinc oxide nanoparticles (ZnO-NPs) from leaves of some plants. Annals of Agricultural Science, Moshtohor.

[ref-46] Francine U, Jeannette U, Pierre R (2015). Assessment of antibacterial activity of neem plant (*Azadirachta indica*) on *Staphylococcus aureus* and *Escherichia coli*. Journal of Medicinal Plants Studies.

[ref-47] García-Cruz L, Salinas-Moreno Y, Valle-Guadarrama S (2012). Betalaínas, compuestos fenólicos y actividad antioxidante en pitaya de mayo (*Stenocereus griseus* H.). Revista Fitotecnia Mexicana.

[ref-48] Gunalan S, Sivaraj R, Rajendran V (2012). Green synthesized ZnO nanoparticles against bacterial and fungal pathogens. Progress in Natural Science: Materials International.

[ref-49] Gupta SC, Prasad S, Tyagi AK, Kunnumakkara AB, Aggarwal BB (2017). Neem (*Azadirachta indica*): an Indian traditional panacea with modern molecular basis. Phytomedicine.

[ref-50] Hansen MB, Nielsen SE, Berg KJ (1989). Re-examination and further development of a precise and rapid dye method for measuring cell growth/cell kill. Journal of Immunological Methods.

[ref-51] Hone DC, Walker PI, Evans-Gowing R, FitzGerald S, Beeby A, Chambrier I, Michael JC, Russell DA (2002). Generation of cytotoxic singlet oxygen *via* phthalocyanine-stabilized gold nanoparticles: a potential delivery vehicle for photodynamic therapy. Langmuir.

[ref-52] Hossain MA, Nagooru MR (2011). Biochemical profiling and total flavonoids contents of leaves crude extract of endemic medicinal plant Corydyline terminalis L. Kunth. Pharmacognosy Journal.

[ref-53] Imbabi T, Hassan A, Ahmed-Farid O, El-Garhy O, Sabeq I, Moustafa M, Mohammadein A, Hassan N, Osman A, Sitohy M (2021). Supplementing rabbit diets with butylated hydroxyanisole affects oxidative stress, growth performance, and meat quality. Animal.

[ref-54] Iravani S (2011). Green synthesis of metal nanoparticles using plants. Green Chemistry.

[ref-55] Itelima J, Nwokedi V, Ogbonna A, Nyam M (2016). Phytochemical screening and antimicrobial activity evaluation of aqueous and ethanolic extracts of the leaf of *Azadirachta indica* Juss (neem) on some microorganisms. World Journal of Microbiology.

[ref-56] Jha AK, Prasad K (2010). Green synthesis of silver nanoparticles using Cycas leaf. International Journal of Green Nanotechnology: Physics and Chemistry.

[ref-57] Kasemets K, Ivask A, Dubourguier H-C, Kahru A (2009). Toxicity of nanoparticles of ZnO, CuO and TiO_2_ to yeast *Saccharomyces cerevisiae*. Toxicology in Vitro.

[ref-58] Kumar VS, Navaratnam V (2013). Neem (*Azadirachta indica*): prehistory to contemporary medicinal uses to humankind. Asian Pacific Journal of Tropical Biomedicine.

[ref-59] Leung Y, Xu X, Ma A, Liu F, Ng A, Shen Z, Gethings L, Guo M, Djurišić A, Lee P (2016). Toxicity of ZnO and TiO2 to *Escherichia coli* cells. Scientific Reports.

[ref-60] Lucantoni L, Yerbanga RS, Lupidi G, Pasqualini L, Esposito F, Habluetzel A (2010). Transmission blocking activity of a standardized neem (*Azadirachta indica*) seed extract on the rodent malaria parasite *Plasmodium berghei* in its vector Anopheles stephensi. Malaria Journal.

[ref-61] Madhavan SA (2021). Phytochemical analysis and anticancer activity of ethanolic extract against A549 human lung cancer cell line *Azadirachta indica*. Journal of Chemistry and Nutritional Biochemistry.

[ref-62] Mahgoub SA, Sitohy MZ, Osman AO (2013). Counteracting recontamination of pasteurized milk by methylated soybean protein. Food and Bioprocess Technology.

[ref-63] Mariappan V, Vellasamy KM, Mohamad NA, Subramaniam S, Vadivelu J (2021). OneHealth approaches contribute towards antimicrobial resistance: Malaysian perspective. Frontiers in Microbiology.

[ref-64] Mthana MS, Mthiyane MN, Ekennia AC, Singh M, Onwudiwe D (2022). Cytotoxicity and antibacterial effects of silver doped zinc oxide nanoparticles prepared using fruit extract of Capsicum Chinense. Scientific African.

[ref-65] Nadhman A, Nazir S, Khan MI, Arooj S, Bakhtiar M, Shahnaz G, Yasinzai M (2014). PEGylated silver doped zinc oxide nanoparticles as novel photosensitizers for photodynamic therapy against Leishmania. Free Radical Biology and Medicine.

[ref-66] Nagano MS, Batalini C (2021). Phytochemical screening, antioxidant activity and potential toxicity of *Azadirachta indica* A. Juss (neem) leaves. Revista Colombiana de Ciencias Químico-Farmacéuticas.

[ref-67] Najafi-Taher R, Ghaemi B, Kharazi S, Rasoulikoohi S, Amani A (2018). Promising antibacterial effects of silver nanoparticle-loaded tea tree oil nanoemulsion: a synergistic combination against resistance threat. Aaps PharmSciTech.

[ref-68] Narde J, Ahmed N, Maiti S, Rajeshkumar S, Ganapathy D (2023). Green synthesis of silver nanoparticles from aloe vera and neem leaf extract and their cytotoxic effect evaluation. Journal of Population Therapeutics and Clinical Pharmacology.

[ref-69] Naser R, Abu-Huwaij R, Al-khateeb I, Abbas MM, Atoom AM (2021). Green synthesis of zinc oxide nanoparticles using the root hair extract of Phoenix dactylifera: antimicrobial and anticancer activity. Applied Nanoscience.

[ref-70] Ordoñez AAL, Gomez JD, Vattuone MA, lsla MI (2006). Antioxidant activities of *Sechium edule* (Jacq.) Swartz extracts. Food Chemistry.

[ref-71] Osman A, Enan G, Al-Mohammadi A-R, Abdel-Shafi S, Abdel-Hameid S, Sitohy MZ, El-Gazzar N (2021a). Antibacterial peptides produced by Alcalase from cowpea seed proteins. Antibiotics.

[ref-72] Osman A, Mahgoub SAM, Wahdan KMM, Ramadan MFJJoFS, Quality F (2019). Antimicrobial and antioxidant influence of Syzygium aromaticum oil supplementation on minced beef quality during cold storage. Journal of Food Safety and Food Quality.

[ref-73] Osman A, Salama A, Mahmoud KEmam, Sitohy M (2021b). Alleviation of carbon tetrachloride-induced hepatocellular damage and oxidative stress in rats by *Anabaena oryzae* phycocyanin. Journal of Food Biochemistry.

[ref-74] Pal S, Mondal S, Maity J, Mukherjee R (2018). Synthesis and characterization of ZnO nanoparticles using Moringa oleifera leaf extract: investigation of photocatalytic and antibacterial activity. International Journal of Nanoscience and Nanotechnology.

[ref-75] Paul R, Prasad M, Sah NK (2011). Anticancer biology of *Azadirachta indica* L (neem): a mini review. Cancer Biology & Therapy.

[ref-76] Rahman ANA, Amer SA, Masoud SR, El-Saber MM, Osman A, Younis EM, Abdelwarith AA, Davies SJ, Khamis T, Ibrahim RE (2023). Neem seed protein hydrolysate as a fishmeal substitute in Nile tilapia: effects on antioxidant/immune pathway, growth, amino acid transporters-related gene expression, and Aeromonas veronii resistance. Aquaculture.

[ref-77] Rajendran R, Mani A (2020). Photocatalytic, antibacterial and anticancer activity of silver-doped zinc oxide nanoparticles. Journal of Saudi Chemical Society.

[ref-78] Ramadan KM, El-Beltagi HS, Bendary ES, Ali HM (2022a). Experimental evaluation of the antioxidant and antitumor activities of thyme and basil essential oils and their phenolic constituents: Theoretical antioxidant evaluation. Chemical and Biological Technologies in Agriculture.

[ref-79] Ramadan KMA, El-Beltagi HS, Mohamed HI, Shalaby TA, Galal A, Mansour AT, Aboul Fotouh MM, Bendary ESA (2022b). Antioxidant, anti-cancer activity and phytochemicals profiling of Kigelia pinnata fruits. Separations.

[ref-80] Ramadan MF, Osman AOM, El-Akad HM (2008). Food ingredients total antioxidant potential of juices and beverages screening by DPPH *in vitro* assay. Deutsche Lebensmittel-Rundschau.

[ref-81] Reda RM, Helmy RM, Osman A, Ahmed FAG, Kotb GA, El-Fattah AH (2023). The potential effect of Moringa oleifera ethanolic leaf extract against oxidative stress, immune response disruption induced by abamectin exposure in *Oreochromis niloticus*. Environmental Science and Pollution Research International.

[ref-82] Sahu N, Soni D, Chandrashekhar B, Satpute D, Saravanadevi S, Sarangi B, Pandey R (2016). Synthesis of silver nanoparticles using flavonoids: hesperidin, naringin and diosmin, and their antibacterial effects and cytotoxicity. International Nano Letters.

[ref-83] Sánchez-Rangel JC, Benavides J, Heredia JB, Cisneros-Zevallos L, Jacobo-Velázquez D (2013). The Folin–Ciocalteu assay revisited: improvement of its specificity for total phenolic content determination. Analytical Methods.

[ref-84] Sangeetha G, Rajeshwari S, Venckatesh R (2011). Green synthesis of zinc oxide nanoparticles by aloe barbadensis miller leaf extract: structure and optical properties. Materials Research Bulletin.

[ref-85] Sani Y, Baburo A (2020). Phytochemical analysis & antioxidant activity of flower & seed oil extract of *Azadirachta Indica* (neem). International Journal for Scientific Research & Development.

[ref-86] Sankhalkar S (2014). Antioxidant enzyme activity, phenolics and flavonoid content in vegetative and reproductive parts of *Moringa oleifera* Lam. American Journal of PharmTech Research.

[ref-87] Sarwar S, Chakraborti S, Bera S, Sheikh IA, Hoque KM, Chakrabarti P (2016). The antimicrobial activity of ZnO nanoparticles against Vibrio cholerae: variation in response depends on biotype. Nanomedicine.

[ref-88] Schirrmacher V (2019). From chemotherapy to biological therapy: a review of novel concepts to reduce the side effects of systemic cancer treatment. International Journal of Oncology.

[ref-89] Schwartz JR, Marsh RG, Draelos ZD (2005). Zinc and skin health: overview of physiology and pharmacology. Dermatologic Surgery.

[ref-90] Selim YA, Azb MA, Ragab I, Abd El-Azim MHM (2020). Green synthesis of zinc oxide nanoparticles using aqueous extract of *Deverra tortuosa* and their cytotoxic activities. Scientific Reports.

[ref-91] Serwecińska L (2020). Antimicrobials and antibiotic-resistant bacteria: a risk to the environment and to public health. Water.

[ref-92] Shrestha P, Adhikari S, Lamichhane B, Shrestha BG (2015). Phytochemical screening of the medicinal plants of Nepal. IOSR Journal of Environmental Science, Toxicology and Food Technology.

[ref-93] Sirelkhatim A, Mahmud S, Seeni A, Kaus NHM, Ann LC, Bakhori SKM, Hasan H, Mohamad D (2015a). Review on zinc oxide nanoparticles: antibacterial activity and toxicity mechanism. Nano-micro Letters.

[ref-94] Sitohy M, Mahgoub S, Osman A, El-Masry R, Al-Gaby A (2013). Extent and mode of action of cationic legume proteins against *Listeria monocytogenes* and *Salmonella Enteritidis*. Probiotics and Antimicrobial Proteins.

[ref-95] Sohail MF, Rehman M, Hussain SZ, Huma ZE, Shahnaz G, Qureshi OS, Qandeel K, Shaper M, Irshad H, Webster TJ (2020). Green synthesis of zinc oxide nanoparticles by Neem extract as multi-facet therapeutic agents. Journal of Drug Delivery Science and Technology.

[ref-96] Suresh J, Pradheesh G, Alexramani V, Sundrarajan M, Hong S (2018). Green synthesis and characterization of zinc oxide nanoparticle using insulin plant (*Costus pictus* D. Don) and investigation of its antimicrobial as well as anticancer activities. Advances in Natural Sciences: Nanoscience and Nanotechnology.

[ref-97] Tayel AA, Moussa S, Opwis K, Knittel D, Schollmeyer E, Nickisch-Hartfiel A (2010). Inhibition of microbial pathogens by fungal chitosan. International Journal of Biological Macromolecules.

[ref-98] Umamaheswari A, Prabu SL, John SA, Puratchikody A (2021). Green synthesis of zinc oxide nanoparticles using leaf extracts of raphanus sativus var. longipinnatus and evaluation of their anticancer property in A549 cell lines. Biotechnology Reports.

[ref-99] Wang C, Hu X, Gao Y, Ji Y (2015). ZnO nanoparticles treatment induces apoptosis by increasing intracellular ROS levels in LTEP-a-2 cells. BioMed Research International.

[ref-100] Wang B, Zhang Y, Mao Z, Yu D, Gao C (2014). Toxicity of ZnO nanoparticles to macrophages due to cell uptake and intracellular release of zinc ions. Journal of Nanoscience and Nanotechnology.

[ref-101] Yadav AR, Mohite SK (2020). Cancer-A silent killer: an overview. Asian Journal of Pharmaceutical Research.

[ref-102] Yu K-N, Yoon T-J, Minai-Tehrani A, Kim J-E, Park SJ, Jeong MS, Ha S-W, Lee J-K, Kim JS, Cho M-H (2013). Zinc oxide nanoparticle induced autophagic cell death and mitochondrial damage *via* reactive oxygen species generation. Toxicology in Vitro.

[ref-103] Zambri NDS, Taib NI, Abdul Latif F, Mohamed Z (2019). Utilization of neem leaf extract on biosynthesis of iron oxide nanoparticles. Molecules.

[ref-104] Zhang L, Jiang Y, Ding Y, Povey M, York D (2007). Investigation into the antibacterial behaviour of suspensions of ZnO nanoparticles (ZnO nanofluids). Journal of Nanoparticle Research.

