# Supplementary material for: Biosynthesis of zinc oxide nanoparticles via neem extract and their anticancer and antibacterial activities
Source: PeerJ. 2024 Jun 25;12:e17588. doi: 10.7717/peerj.17588 (PMC11212640; doi:10.7717/peerj.17588)
Supplement: Supplemental Information 2 [file peerj-12-17588-s002.docx]

**Rebuttal letter for adding author**

We (All authors) are the participating authors in this manuscript agree and confirm the authorship of Dr. Hind Althagafi according to the CRediT author contribution.

Due to the substantial contribution of Dr. Althagafi in preparing the revised version of this manuscript, which includes addressing the comments from the reviewers regarding the interpretation of our data which is related to the nanotechnology and plant biological activity as she has Ph.D in this field form United Kingdom. Also, revising the manuscript critically for important intellectual content and the language improvement.

Dr. Althagafi resolved the reviewer’s comments such as Improve the ZnONPs syntheses section in both chemical and green synthesis, Validity of the findings, Novelty of your study, the chemical and biological synthesis part of zinc oxide in the methods, comparing our results with other findings, add product identifiers like product codes, equipment details, re-write results and discussion section and improve the figures resolutions

All parts of the manuscript handled by Dr was highlighted with green highlight color for your consideration.

Therefore, we hope that the editors and reviewers of your respected journal will approve the addition to complement the efforts of our research team by publishing our manuscript.

Therefore, in the revised manuscript, we have decided to include her as a co-author of the paper. All co-authors approve of the change in the authorship in the journal systems.

Corresponding Author:

[Hossam El-Beltagi](https://sciprofiles.com/profile/952105)

[helbeltagi@kfu.edu.sa](mailto:helbeltagi@kfu.edu.sa); [https://orcid.org/0000-0003-4433-2034](https://www.scopus.com/redirect.uri?url=https://orcid.org/0000-0003-4433-2034&authorId=26656704600&origin=AuthorProfile&orcId=0000-0003-4433-2034&category=orcidLink)

Ali Osman

[aokhalil@zu.edu.eg](mailto:aokhalil@zu.edu.eg); <http://orcid.org/0000-0001-7174-0207>
